# The Impact of Hydrostatic Pressure on the Structural, Mechanical, Thermal, and Optoelectronic Characteristics of the RbV_3_Sb_5_ Kagome Compound: Ab initio Approach

**DOI:** 10.1002/open.202400291

**Published:** 2024-10-21

**Authors:** Prianka Mondal, Md. Raihan Islam, Mst. Shamima Khanom, Farid Ahmed

**Affiliations:** ^1^ Department of Physics Dhaka University of Engineering and Technology (DUET) Gazipur Gazipr-1707 Bangladesh; ^2^ Department of Physics Jahangirnagar University Savar Dhaka-1342 Bangladesh

**Keywords:** RbV_3_Sb_5_ kagome, Mechanical and thermal properties, DFT, Phonon property, Optoelectronics

## Abstract

We studied the RbV_3_Sb_5_ kagome compound's structural, mechanical, thermal, and optoelectronic properties. Mulliken and Hirshfeld population analysis found ionic and covalent connections in RbV_3_Sb_5_. The Born stability criterion shows that pure RbV_3_Sb_5_ is mechanically stable. The precise measurement of 3.96 indicates that our sample has higher machinability at 20 GPa. Low anticipated hardness of RbV_3_Sb_5_ suggests it can be used as a soft solid lubricant. Hardness ratings rise with pressure, however there are exceptions. Pressure causes large nonmonotonic changes in RbV_3_Sb_5_′s anisotropic characteristics. A comparable 20 GPa Zener anisotropic value, RbV_3_Sb_5_ has the highest. The structure's projected Debye temperature at 0 GPa is 284.39 K, indicating softness. Dispersion curves with negative frequencies suggest ground state structural dynamical instability. The structure has no negative‐energy phonon branches under 10 GPa stress. From band structure and density of state analysis, the structure behaves metallically under hydrostatic pressure. Also, the structure has maximal ultra‐violet conductivity and absorption. The absorption coefficient, conductivity, and loss function plots show uniform patterns at all pressures. As pressure rises, these graphs’ peaks blue shift.

## Graphical abstract

We investigated the RbV_3_Sb_5_′s several properties. Pure mechanically stable structure has ionic and covalent bonds. Low predicted hardness means it can be a soft solid lubricant. RbV_3_Sb_5_′s anisotropy changes significantly under pressure. Dispersion graphs indicate structural dynamical instability in ground state. Band structure and DOS show metallic behavior under hydrostatic pressure. Additionally, the structure has maximum UV conductivity and absorption.

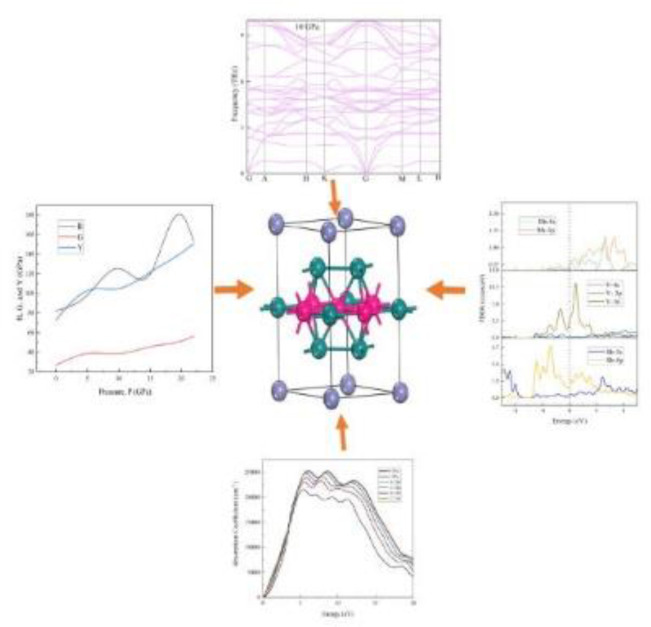

## 1 Introduction

Kagome materials, characterized by their geometric frustration and distinctive band structure, offer a valuable platform for the exploration, categorization, and comprehension of linked electronic phases in quantum matter.[^1^, ^2^, ^3^, ^4^, ^5^] Kagome metals may stabilize novel coupled and topological electronic states. The kagome lattice's electron filling can cause bond density wave order, charge fractionalization, spin liquid states, charge density waves, including superconductivity. The kagome structural motif additionally permits electronically nontrivial structures with Dirac cones and flatbands, which can cause significant correlation effects and topological states. Kagome compounds’ magnetic order maintains distinct quantum anomalous hall behaviors. Electron interactions may also induce topological insulating phases in certain settings. An electrical instability on a two‐dimensional kagome lattice that creates a superconducting ground state is needed. A recent study unveiled a novel category of layered kagome metals that exhibit crystallization in the AV_3_Sb_5_ structure, with A representing K, Rb, or Cs.[^6^, ^7^] The AV_3_Sb_5_ family of kagome metals, display a variety of intriguing phenomena, including significant anomalous Hall conductivity, charge order, orbital order, and potential unconventional superconductivity.[^8^, ^9^] Such compounds are two‐dimensional metals with high mobility. They display correlated effects and electrical variables may destroy symmetry. One form, KV_3_Sb_5_, has Dirac semimetal characteristics, according to recent research. Notably, it has a very strong anomalous hall effect even without long‐range magnetic order.^[10]^ Like Kagome several types of structures which have transition metals were studied previously using first principles.[^11^, ^12^, ^13^, ^14^, ^15^, ^16^]

The initial isolation of the prototype KV_3_Sb_5_ occurred in powdered form, and its structure was determined through the application of charge‐flipping techniques on data obtained from powder diffraction. One investigation revealed that only KV_3_Sb_5_, RbV_3_Sb_5_, and CsV_3_Sb_5_ exhibit crystallization in the KV_3_Sb_5_ prototype. Based on the powder diffraction data, they observed that KV_3_Sb_5_, RbV_3_Sb_5_, and CsV_3_Sb_5_ exhibit crystallization in the hexagonal P6/mmm space group. The vanadium sub lattice notably exhibits a structurally flawless Kagome net. KV_3_Sb_5_ represents a novel Kagome prototype structure and serves as a compact and uncomplicated illustration of a Kagome lattice.^[10]^ The kagome material CsV_3_Sb_5_, which was discovered this past year, was determined to be a quasi‐2D kagome superconductor with a transition temperature Tc of approximately 2.3 K.^[6]^ Despite the comparable physical features exhibited by all three AV_3_Sb_5_ (A=K, Rb, Cs) compounds, CsV_3_Sb_5_ is presently the subject of extensive research, perhaps because to its superior Tc value.^[17]^ Furthermore, superconductivity was discovered in the complete range of compounds KV_3_Sb_5_ (with a critical temperature of around 0.93 K) and RbV_3_Sb_5_ (with a critical temperature of approximately 0.75 K). This discovery has sparked significant research endeavors in this particular domain. Therefore, all AV_3_Sb_5_ compounds in the material family exhibit superconductivity at low temperatures. These materials have recently demonstrated superconductivity at low temperatures and a unique charge order at high temperatures, indicating a link to the fundamental topological properties of the band structure.[^18^, ^19^]

We conducted a comprehensive study on the magneto resistance and hall resistivity of single crystals of the kagome superconductor RbV_3_Sb_5_. The crystals exhibit distinct quantum oscillations with multiple frequencies. The analysis of oscillations uncovers the presence of three bands that possess a two‐dimensional nature and accommodate light quasi particles with significant effective mass and nontrivial Berry phase.^[20]^ Single crystals of RbV_3_Sb_5_ exhibit a superconducting transition at a critical temperature of approximately 0.92 K. Resistivity, magnetization, and heat capacity tests suggest that it displays unusual features at a temperature of T*∼102–103 K, which may be linked to the development of a charge ordering state.

One study relating pressure effect showed that the charge order of CsV_3_Sb_5_ is diminished when subjected to a moderate pressure (1.4 GPa<pressure<1.6 GPa), whereas the superconducting transition temperature Tc is optimized. The critical temperature (Tc) is initially weakened as pressure increases, eventually reaching a low at approximately 14.3 GPa. It then shows another peak at roughly 22.8 GPa, indicating the existence of a second region of superconductivity.^[21]^ Despite their embedded structure, powders, single crystals, and densified pellets of KV_3_Sb_5_, RbV_3_Sb_5_, and CsV_3_Sb_5_ exhibit remarkable stability at temperatures of 400 °C, 600 °C, and 800 °C.^[10]^

## Computational Method

The RbV_3_Sb_5_ kagome structure was optimized using the Cambridge Serial Total Energy (CASTEP) technique for density‐functional theory (DFT) simulations.[^22^, ^23^] Then, under varying hydrostatic pressures, the optimized crystals’ optical, structural, mechanical, phonon, thermal, and electro‐magnetic properties were computed. Specifically, the Kohn‐Sham equations are solved iteratively and independently at pre‐set kinetic cut‐off energies by the CASTEP code. Afterwards, it makes an approximation of the many‐electron system's ground state energy. The ultrasoft pseudopotential was used to measure the electron‐ion interaction in the present study. The impact of tightly bound core electrons on the essential nature of the ground state of the structure is demonstrated by this interaction.^[24]^ Ultrasoft pseudopotential (USP) calculations can make use of the plane‐wave basis set's lowest cutoff energy.^[25]^ After several threshold energies converged, we settled on 400 eV for our computations. A version of the generalized gradient approximation (GGA) exchange‐correlation functional called Perdew‐Burke‐Erzenhof for solids (PBEsol) was employed for optimizing the crystal structure and calculating other parameters.^[26]^ We also used HSE06 hubrid functional for calculating electronic property. To assess the structural parameters, we employed the BFGS (Broyden‐Fletcher‐Goldfarb‐Shanno) minimization code.^[27]^ We adhered to certain preset conditions, such as a residual force of less than 0.05 eV/Angstrom and an energy change per atom of less than 1×10^−6^ eV. Atomic stress was limited to 0.1 GPa and atomic displacement to 0.002. We did not consider the spin‐orbit coupling in the calculations.

In the Brillouin zone of the unit cell, we utilized an 11×11×6 Monkhorst‐Pack grid^[26]^ to generate a consistent k‐points grid in the reciprocal space along all three axes. Denser k‐point meshes, 23×23×12, are required for the fermi surface computations of RbV_3_Sb_5_.

This study presents and analyses the optical conductivity σ(ω), the reflectivity, R(ω), the refractive index n(ω) and the extinction coefficient k(ω).[^28^, ^29^] The complex component of the dielectric function governs the optical properties of a material. The formula ϵ(ω)=ϵ_1_(ω)+iϵ_2_(ω) is called the Kramer‐Kronig relationship in mathematics.^[30]^ The following is the representation of the imaginary part of the dielectric function,$\epsilon_{2}\left(\omega \right)$ in this study:

$$\epsilon_{2}\left(\omega \right)=\left(\frac{4\pi^{2}e^{2}}{m^{2}\omega^{2}}\right)\;\sum_{ij}\int {\left\langle i\left|M_{d}\right|j\right\rangle }^{2}f_{i}\left(1-f_{i}\right)\delta \left(E_{f}-\;E_{i}-\;\omega \right)d^{3}k$$

With *i* standing for the initial state and *j* for the final state, we have. $f_{i}$ denotes the fermi distribution function for *i*‐th state, $M_{d}$ denotes the dipole matrix, and the energy of the electron in the *i*‐th state is represented by $E_{i}$ . Using Kramers‐Kroning relation, it is possible to deduce the real portion of the dielectric function from its imaginary portion, and can be represent as:

$$\epsilon_{1}\left(\omega \right)=1+\;\frac{2}{\pi }P\int_{0}^{\infty }\frac{\omega^{'}\epsilon_{2}\left(\omega^{'}\right)d\omega^{'}}{\left({\omega^{'}}^{2}-\;\omega^{2}\right)}$$

Let P denote the principal value of the integral. To accurately compute significant optical properties, one must possess comprehension of the dielectric tensor's real and imaginary constituents.

## 2 Results and Discussion

### 2.1 Structural Parameters

**Figure 1:**
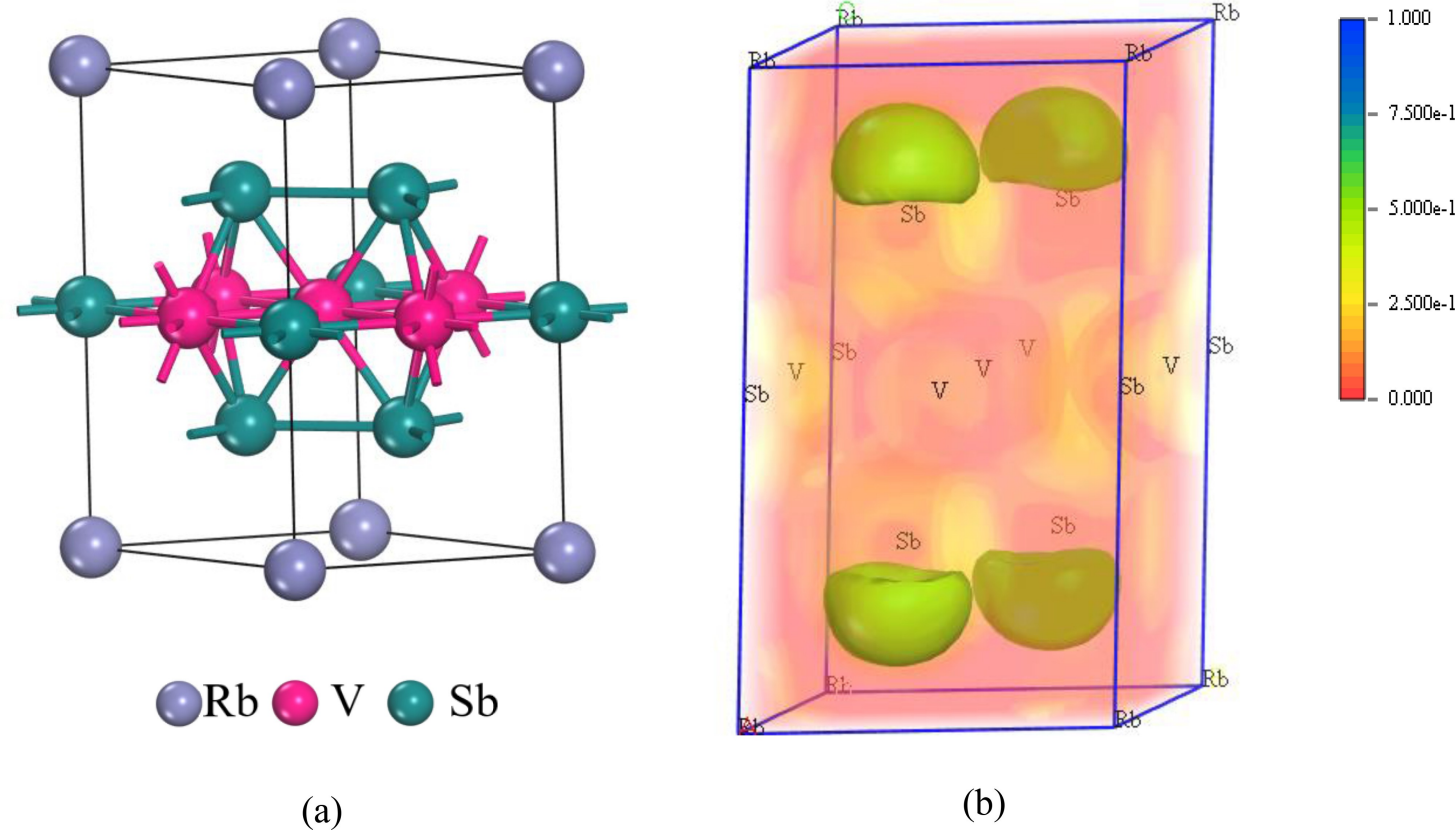
(a) Crystal structure and (b) Electron localization function (ELF) of hexagonal RbV_3_Sb_5_ compound.

RbV_3_Sb_5_ material has a vanadium‐based quasi‐2D Kagome lattice that is structurally impeccable. Alkali metal cations are distributed between V_3_Sb_5_ slab layers in the compounds. One of the Sb sublattices has two graphene‐like layers on either side of the Kagome lattice. Calculations show that alkali metal cations have low binding strength to V_3_Sb_5_ slabs and can easily separate from the lattice. It has negatively charged V−Sb layers separated by positively charged alkali metal cations (Figure 1). Interestingly, the vanadium sublattice has a faultless Kagome network. We can break the RbV_3_Sb_5_ structure into atomic sublattices Rb, V, Sb1, and Sb2 to see it differently. It indicates two types of antimony (Sb) sites. Sb1 atoms are at the cores of vanadium (V) hexagons. However, the Sb atoms at the Sb2 site are below and above the centres of V atom triangles, forming hexagonal layers like graphene.

Electron localization function (ELF) in CASTEP is estimated solely for valence electrons. The blue color in the ELF maps according to our label suggests a higher electron density. Notably, the green color (Figure 1) primarily surrounds the Sb atoms, indicating a concentration of localized electrons in particular locations. This finding reveals important details regarding the electron‐rich nature of Sb‐containing bonds. In contrast, the red color on the ELF maps indicates places with lower electron density, implying a higher degree of electron delocalization.

We optimized the structure for different configurations, such as, Non‐Magnetic configuration (NM), Ferromagnetic configuration (FM) as well as Anti‐Ferromagnetic (AFM) configuration. We obtained different ground state energies of the pristine structure at different configurations (Table 1). The fact is, the obtained minimum ground state energy is −7336.332 eV for NM configuration. That means the structure is more stable in this configuration which indicates the structure be non‐magnetic at 0 K temperature which matches with previous study.^[31]^

**Table 1:** Calculated ground state energies for different configurations.

NM Configuration	FM Configuration	AFM Configuration
−7336.332 eV	−7336.331 eV	−7336.237 eV

**Figure 2:**
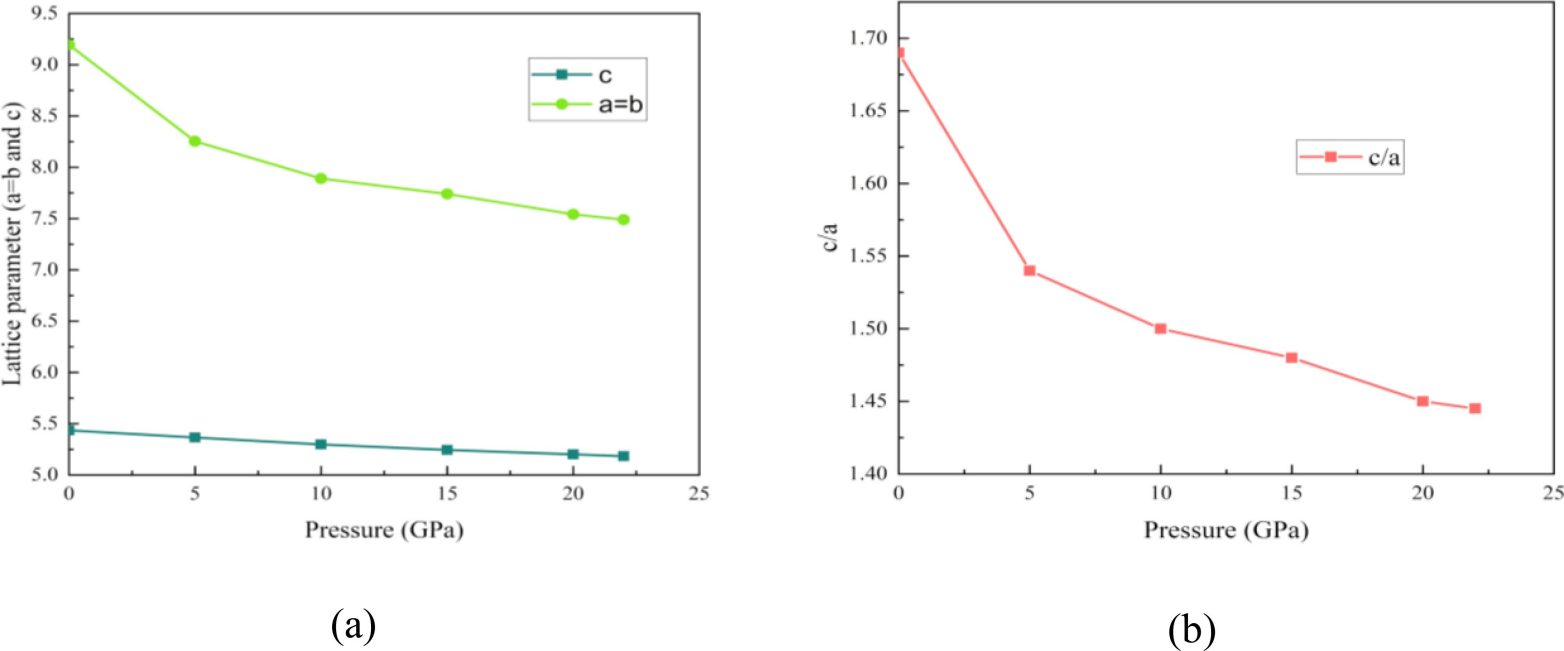
Variation of lattice parameters and c/a ratio with hydrostatic pressure up to 22 GPa.

Due to the P6/mmm space group's high symmetry and small unit cell, the RbV_3_Sb_5_ prototype has just three structural degrees of freedom: a, and c lattice parameters and Sb2′s z‐coordinate. The unit cell consists total nine atoms including one Rb atom, three V atoms and five Sb atoms. RbV_3_Sb_5_ expands in the c direction due to its larger ionic radii, increasing the distance between V−Sb slabs. The Figure 1 shows the crystal structure of RbV_3_Sb_5_. In another study, XRD measurement of a single RbV_3_Sb_5_ crystal showed that its surface is parallel to the (00 l)‐plane. The c‐axial lattice constant is estimated at 9.192 Å.^[32]^ Table 2 tabulates the value of lattice parameters, volumes, ground state energy and formation energy with increasing hydrostatic pressure. The lattice parameters of pristine structure closely match with previous study. We can see the values of ground state energy increase with increasing trend of the pressure. On the other hand, due to pressure the atoms of the structure become close and so the volume of the structure decreases with the increment of the pressure.^[33]^ Figure 2 also suggests this, as both the lattice parameters (a=b and c) and the ratio of c and a decrease with pressure.

**Table 2:** Under various hydrostatic pressures (GPa), the calculated and experimental lattice constants (Å), equilibrium volume V_0_ (Å^3^), total number of atoms in the cell, and chemical unit x of RbV_3_Sb_5._

P	*a=b*	*c*	*c/a*	*V_0_*	No. of atoms	*x*	Remarks	Ground State Energy	Formation energy
0	5.433	9.192	1.69	235.032	9	1	This work	−7336.332	−76.07
5	5.359	8.225	1.53	204.615				−7335.961	−80.65
10	5.294	7.926	1.5	192.412				−7335.389	−80.09
15	5.236	7.712	1.47	183.14				−7334.67	−79.37
20	5.188	7.544	1.45	175.845				−7333.872	−78.57
22	5.17	7.50	1.45	173.60				−7333.577	−78.28
0	–	9.114					^[33]Theo.^		

The justification for using formation energy as a metric to determine thermodynamic stability lies in its ability to quantify the energy exchange that occurs throughout a chemical reaction. The equation for formation energy is expressed as,

$$E_{Formation}=\;E_{total}--E_{Rb}{-3E}_{V\;}{-5E}_{Sb\;}$$

Here, $E_{total},\;E_{Rb}{,\;E}_{V\;}{and\;E}_{Sb\;}$ represent total energy of the structure, energies of isolated Rb, V and Sb atoms respectively. The obtained values are tabulated in Table 2 and all the values are negative.

The Mulliken, and Hirshfeld electric charges as well as charge spilling parameter (CSP) are shown in Table 3. Mulliken bond population analysis (MPA)^[34]^ can determine structural hardness, bond type, and atom electric charge contribution. The study found that the material had the highest population of V atoms compared to other species. The total charge of Sb1 is somewhat higher than Sb2. According to Table 3, all of the atoms’ formal ionic charges were different from what would be expected in a completely ionic condition. Atoms have positive and negative charges, which represent the amount of electrons provided and taken, respectively. It follows that the V−Rb and V−Sb atoms must have an ionic contribution. This also suggests covalent connection between Rb−Rb, Rb−Sb, and Sb−Sb atoms since V atoms transfer more electrons than other species. Ionic and covalent bonds exist in RbV_3_Sb_5_′s bonding. Despite its popularity, the MPA may provide results that contradict the chemical state. Hirshfeld population analysis (HPA) is more reliable so we calculated this.^[35]^ The HPA produced far lower atomic charges per atom than the MPA. A species’ EVC is the difference between an atom's formal ionic charge and its Mulliken or Hirshfeld charge.^[36]^ Additionally, it reveals covalent, ionic, or mixed bond strength. Any positive EVC number suggests better covalence, while zero denotes complete ionic bonding. The predicted EVCs for each species are in Table 3. A nonzero EVC for each atom indicates strong covalent bonding in the solution. However, the HPA and MPA agree on electron transit from Rb and Sb to V atoms. The system's most positively charged Rb atoms and negatively charged V atoms are strongly bonded. Compare the Mulliken charge's EVC to the Hirshfeld charge's are small, and all effective valences predict a greater covalency.

**Table 3:** The charge spilling parameter (%), orbital charges (electron), atomic Mulliken charges (electron), formal ionic charge, EVC (electron), and Hirshfeld charge (electron) of RbV_3_Sb_5_ in its ground state.

Charge Spilling	Species	Mulliken Atomic Population				Mulliken Charge (e)	Formal Ionic Charge	EVC	Hirshfield Charge	EVC
		s	p	d	Total					
0.37	Rb	2.17	5.91	0	8.08	0.92	+1	0.08	0.19	0.81
	V	2.53	6.89	3.99	13.41	−0.41	+3	2.59	−0.12	2.88
	Sb1	1.71	3.29	0	4.99	0.01	0	0.01	0.01	0.01
	Sb2	1.51	3.18	0	4.69	0.31	0	0.31	0.12	0.12

### 2.2 Mechanical Properties:

The physical qualities of a structure are determined by its elastic properties. When it comes to characterize the mechanical properties of materials and determining the threshold for externally imposed mechanical stress in real‐world applications, the second‐order elastic constants of materials are essential and important variables to consider. One can utilize the elastic constants of a material to determine its stability, plasticity, stiffness, brittleness, ductility, chemical bonding, thermal characteristics, and anisotropy. These qualities can be determined by using the elastic constants. The determination of elastic constants is based on the correlation between stress and strain.

To calculate those characteristics in different crystal structures, one must obtain the values of several elastic stiffness constants. The hexagonal crystal system requires six distinct elastic stiffness constants, namely C_11_, C_12_, C_13_, C_33_, C_44_, and C_66_. The Born‐Huang criteria state that the conditions of mechanical stability at equilibrium are subject to vary depending on the symmetries of the crystal. Because of its hexagonal crystal shape, the RbV_3_Sb_5_ molecule is able to fulfill the Born stability requirement; this is the case.

$$c_{11}-\left|c_{12}\right|>0$$

$$C_{33}\left(C_{11}+C_{12}\right)-2C_{13}^{2}>0$$

$$C_{44}>0$$

The constants of elasticity for RbV_3_Sb_5_ under different pressures are presented in Table 4. The stability requirements for such crystal structure when subjected to pressure are; 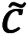
_11 >_ | 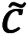
_12_|_,_ 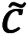
_33_( 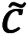
_11_ + 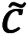
_12_) > 2 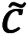 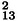 ,⋅ 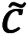
_44_ > 0 where, 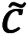
_*ii*_ = c_*ii*_ − P(*i* = 1,3) and 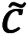
_1*i*_ = c_1*i*_ + P(*i* = 2,3). According to these stability criterion, RbV_3_Sb_5_ shows stability for all hydrostatic pressure. Along the [001] direction, $C_{44\;}$ resents the shearing stress in the (010) plane is quantify the reaction to a shear stress.^[37]^ Additionally the machinability indices and stiffness of materials are directly connected to this elastic constant. It is also obtain from Table 4 that the values of $C_{44}$ at different pressure than that of $C_{66}$ , which infers that shearing is more dominant along the (100) plane as compare to that of along the (001) plane. Such variation in shear distortions can be attributed to the distinct variations in atomic structure and their interplay. Moreover, Strong atomic bonding along the c‐axis compared to the a‐axis is shown by confirm by the $C_{11}>C_{33}$ as we find from the Table 4. This means that along [100], RbV_3_Sb_5_ is more amenable to compression.

**Table 4:** Computed values of elastic constants *C_ij_* (GPa), machinability index $\mu_{M}$ , and isotropic bulk modulus *B* (GPa) of RbV_3_Sb_5_ under different hydrostatic pressures (GPa).

P (GPa)	C_11_	C_33_	C_12_	C_44_	C_13_	C_66_	$\mu_{M}$	B (GPa)	Remarks
0	115.59	141.69	59.56	22.99	63.49	28.01	3.58	82.38	This work
5	171.04	155.82	80.89	28.03	57.71	45.07	3.51	98.45	
10	204.53	204.44	85.66	20.29	86.39	59.43	6.19	125.52	
15	222.30	174.63	95.33	28.12	71.27	63.48	4.27	120.17	
20	267.58	16.50	119.09	21.01	48.17	74.24	8.58	180.17	
22	290.06	208.32	136.27	34.80	87.23	76.89	4.39	152.81	

For engineering materials, a useful parameter known as machinability index indicates how readily it may perform when subjected to different cutting operations including milling, drilling, or grinding. This index is useful for determining the usability of machining equipment and techniques on a certain material. This factor is affected by mechanical qualities, chemical composition, microscopic structure, and other features of the material. There is a direct correlation between the machinability index and the plasticity of an industrial substance, which increases as the index rises.^[38]^ The machinability index of a material can be expressed as.^[39]^

$$\mu_{M}=\;\frac{B}{C_{44}}$$

The efficiency of our sample is greater at 20 GPa when compare to other pressure conditions listed in Table 4. Additionally, the solids that are easy to machine also have great lubricating qualities, which means they have reduced friction values, and feed forces. For evaluation purposes, we list available experimental and theoretical $\mu_{M}$ values in literature for materials like diamond, silver, gold, and CsV_3_Sb_5_[^40^, ^41^, ^42^] in Table 5. Compared to other materials, RbV_3_Sb_5_ has superior lubricating capabilities, might be useful in the production sector. Our sample exhibits a greater machinability at a steady pressure of 20 GPa, with a value of 8.58.

**Table 5:** The calculated machinability of RbV_3_Sb_5_, in comparison to the experimental values of diamond silver, CsV_3_Sb_5_ and gold.

Material	Diamond	Ag	CsV_3_Sb_5_	RbV_3_Sb_5_	Au
$\mu_{M}$	0.772	2.175	2.936	3.58	4.168
Remark	^[40]Expt.^		^[41]Theo.^	This work	^[41]Expt.^

Based on the second‐order elastic constants *C_ij_*, the Voigt‐Reuss‐Hill (VRH) formula[^43^, ^44^] was used to compute different elastic moduli are listed in Table 5. Using these widely‐known equations, we were able to determine the VRH bulk, shear, and Young's moduli of our sample.^[45]^

$$B_{V}=\frac{\left[C_{11}+C_{22}+C_{33}+2\left(C_{12}+C_{13}+C_{23}\right)\right]}{9}$$

$$G_{V}=\frac{\left[C_{11}+C_{22}+C_{33}+3\left(C_{44}+C_{55}+C_{66}\right)-\left(C_{12}+C_{13}+C_{23}\right)\right]}{15}$$

$$B_{H}=\frac{B_{V}+B_{R}}{2}\;;\;G_{H}=\frac{G_{V}+G_{R}}{2}$$

**Figure 3:**
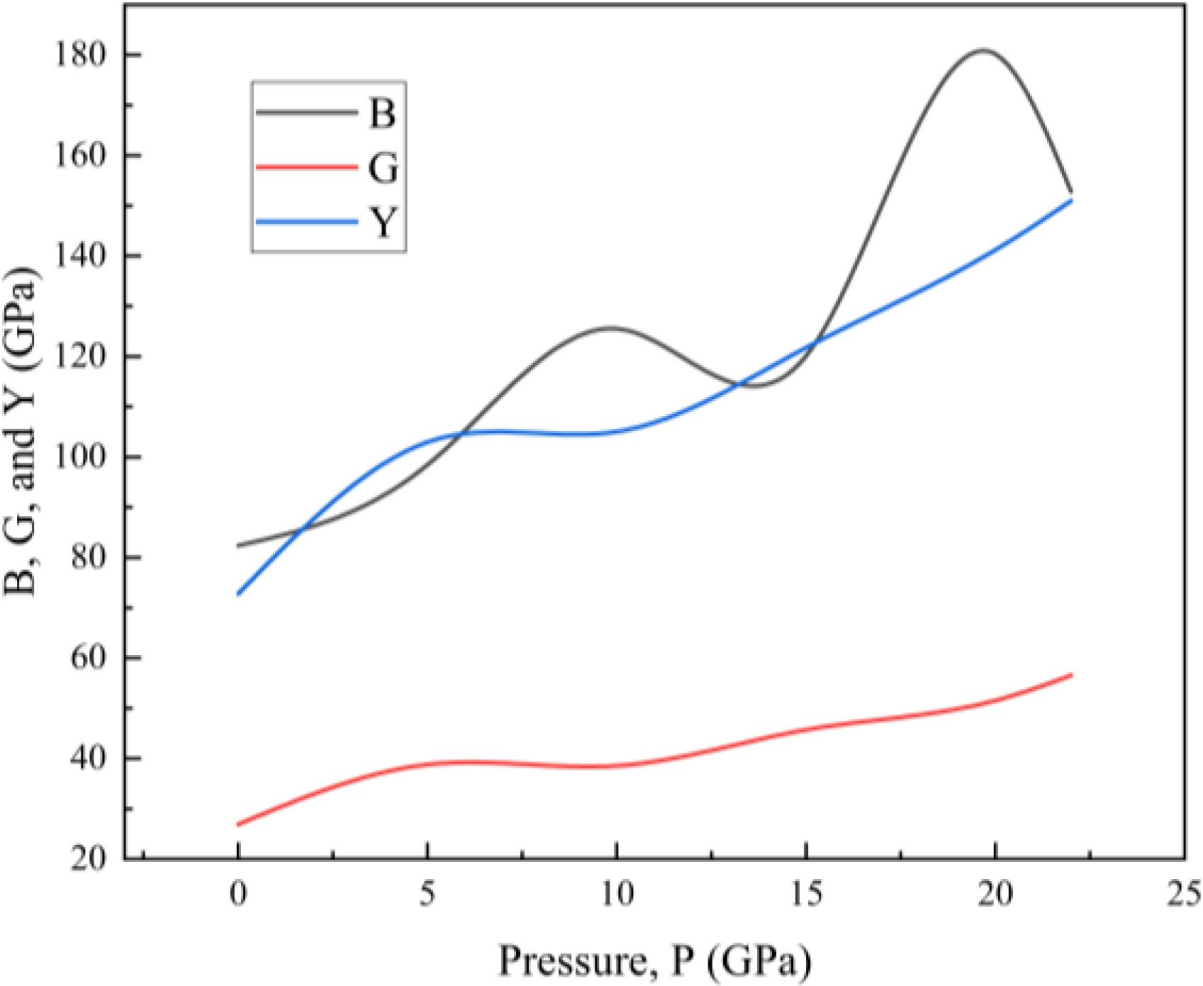
Bulk, Shear, and Young modulus of RbV_3_Sb_5_ as a function of pressure.

The bulk modulus (B) is a way to quantify how resistant a material is to uniform deformation. It is an essential factor to consider when designing structures to resist variations in stress or external influences. Every pure metal has a B value that is directly proportional to its rupture strength. The response of materials to stresses that induce the sliding of parallel layers relative to each other can be better understood using the knowledge of shear modulus (G). A greater shear modulus indicates that the material is less pliable and more inflexible. Directional bonding between atoms is more apparent as G grows in value.^[46]^ A rise in hydrostatic pressure causes both of these parameters of our sample to have greater values. The pressure variation of elastic moduli B, G, and Y is shown in Figure 3. The lower G number than B in Table 6 indicates that its mechanical durability will be overshadowed by its plasticity. The ductility nature of our pristine compound is confirmed by the Pugh ratio values which is 0.33. The G/B values is fluctuates with the changes of hydrostatic pressure. The strain (ϵ ) at fracture, according to Pugh, may be found by integrating the function $\epsilon \propto {\left(\frac{G}{B}\right)}^{2}$ , and hence the materials exhibiting a low Pugh ratio exhibit a significant strain upon fracture.

**Table 6:** The calculated isotropic bulk modulus *B* (GPa), shear modulus *G* (GPa), Young's modulus *Y* (GPa), Pugh's indicator *G*/*B*, Poisson's ratio *σ*, compressibility *β* (1/GPa), and Cauchy pressures (GPa) of RbV_3_Sb_5_ under different hydrostatic pressures (GPa).

P	B			G			Y	$\frac{B}{G}$	$\frac{G}{B}$	σ	β	$C_{p}^{a}$	$C_{p}^{b}$
	BV	BR	BH	GV	GR	GH							
0	82.87	81.89	82.38	27.23	26.59	26.91	72.81	3.06	0.33	0.35	0.012	40.46	31.55
5	98.95	97.96	98.45	40.34	37.33	38.83	102.96	2.54	0.39	0.33	0.010	29.68	35.82
10	125.60	125.45	125.52	43.67	33.52	38.59	105.03	3.25	0.31	0.36	0.007	66.1	26.23
15	121.67	118.67	120.17	49.37	42.11	45.74	121.78	2.63	0.38	0.33	0.008	43.15	31.85
20	180.36	179.97	180.17	55.68	47.44	51.56	141.22	3.49	0.29	0.37	0.006	27.16	44.85
22	156.65	148.96	152.81	61.15	51.95	56.55	151.01	2.70	0.37	0.34	0.0067	52.43	59.38

Another important parameter is the Poisson's ratio which describe the response of the material when subjected to a load. According to reference,^[47]^ ductile solids are those with σ>0.26 while brittle solids are those with σ<0.26 . Therefore, the ductility nature of our compound again confirmed by its σ values at all examined pressure. According to references,^[48]^ the range of values for σ for central‐force solids is between 0.25 and 0.50. Hence, our material is likely to be stable, ductile, and equipped with a core inter‐atomic force. The ideas of Cauchy pressure pertain to the internal forces and pressures experienced by a material. It can further enhance the ductility of RbV_3_Sb_5_. The Cauchy pressure for the (100) and (001) planes is represented by $C_{p}^{a}$ and$C_{p}^{b}$ , respectively. Table 6 summarizes the calculated Cauchy pressures of our compound for these two planes. A positive Cauchy pressure is displayed by RbV_3_Sb_5_ refers metallic bonds.

How well a material resists deformation under a given stress is the determining factor in its Vickers hardness, a measure of its hardness. In the realm of metallurgy, for example, tensile strength and fatigue resistance are two characteristics that might be associated to hardness. A number of factors, including the material's heat treatment, contaminants, and the testing circumstances, might affect the hardness results. Table 7 displays the predicted hardness values macroscopic models[^49^, ^50^] of the substance. It has been observed that the calculated hardness of RbV_3_Sb_5_ is quite low. We may infer from the low hardness value that our compound exhibits a substantial friction coefficient when lubricated with oil. Also the RbV_3_Sb_5_ can be used as a soft solid lubricant.^[51]^ The overall trend of the values of hardness is increases with increasing pressure though there are certain fluctuations.

**Table 7:** The calculated values of hardness (GPa) of RbV_3_Sb_5_ at different hydrostatic pressures (GPa).

*P (GPa)*	*H_1_*	*H_2_*	*H_3_*	*H_4_*	*H_5_*	*H_6_*	*H_7_*	*H_8_*
0	7.93	4.42	3.97	4.62	1.86	2.98	2.64	0.751
5	9.48	6.25	5.73	6.54	3.97	1.87	6.68	2.65
10	12.09	6.38	5.69	6.67	3.93	4.57	4.52	1.31
15	11.57	7.39	6.75	7.73	5.19	3.82	2.83	3.03
20	17.35	8.57	7.61	8.97	6.22	3.36	16.49	1.72
22	14.71	9.17	8.34	9.59	7.10	6.91	6.79	3.62

### 2.3 Elastic Anisotropy

About every crystalline structure that has been discovered has anisotropic characteristics. The kind and amount of anisotropic affect the production of small cracks in solids, the motion of cracks, and the appearance of plastic deformations in crystals. Anisotropy in atomic bond strengths on different planes is defined, for instance, by shear anisotropic factors. Therefore, in order to comprehend their durability and applications in various outside environments, it is essential to know how to accurately calculate the elastic anisotropy factors of RbV_3_Sb_5_.

In order to determine the shear anisotropy on various crystalline planes and in various orientations, three variables must be considered.^[46]^ In the {100} shear plane, the shear anisotropy factor is between the ⟨011⟩ and ⟨010⟩ directions is,

$$A_{1}=\;\frac{4C_{44}}{C_{11\;}+\;C_{33\;}-\;2C_{13}}$$

The shear anisotropic factor is for {010} shear planes that lie between the ⟨101⟩ and ⟨001⟩ directions is,

$$A_{2}=\;\frac{4C_{55}}{C_{22}\;+\;C_{33}\;-\;2C_{23}}$$

Between the directions of ⟨110⟩ and ⟨010⟩, the shear anisotropy factor is for {001} shear planes,

$$A_{3}=\;\frac{4C_{66}}{C_{11}+C_{22}-2C_{12}}$$

The computed shear anisotropic factors for RbV_3_Sb_5_ are presented in Table 8. All three parameters must be set to one for isotropic crystals. At a pressure of 0 GPa, the calculated values of A_1_ (=A_2_) and A_3_ are 0.706 and 1.0, respectively. These values are highest among all examined pressure. On the {001} planes, the material exhibits shear isotropy. The anisotropic degree of a crystal is non‐zero. The predicted values of these parameters indicate that the RbV_3_Sb_5_ is somewhat anisotropic, and here are also notable nonmonotonic variations with pressure.

**Table 8:** Shear anisotropic factors (*A*_1_ *, A*_2_ and *A*_3_), the universal anisotropy index *A* ^*U*^, equivalent Zener anisotropy measure *A* ^*eq*^, anisotropy in shear *A_G_* (or *A* ^*C*^), anisotropy in compressibility *A_B_*, universal log‐Euclidean index *A* ^*L*^, linear compressibilities ($\beta_{a}$ and $\beta_{a}$ ) (TPa^−1^) and their ratio ($\beta_{c}$ /$\beta_{a}$ ) for RbV_3_Sb_5_ at *T*=0 K and at different pressure.

P (GPa)	*A_1_=A_2_*	*A_3_*	*A* ^*U*^	*A* ^*eq*^	*A* ^*G*^	*A* ^*B*^	*A* ^*L*^	$\beta_{a}$	$\beta_{C}$	$\beta_{c}$ /$\beta_{a}$
0	0.706	1.0	0.132	1.391	0.019	0.006	0.063	0.005	0.003	0.624
5	0.53	1.0	2.63	3.97	0.04	0.005	0.342	0.012	0.017	1.42
10	0.3444	1.0	6.14	7.02	0.13	0.001	0.39	0.011	0.0116	1.05
15	0.442	1.0	3.01	4.29	0.08	0.012	0.91	0.008	0.014	1.75
20	0.45	1.0	7.44	8.11	0.07	0.001	1.87	−0.002	0.021	−10.5
22	0.43	1.0	3.36	4.6	0.08	0.025	0.87	0.006	0.011	1.83

The universal anisotropy index *A* ^*U*^, equivalent Zener anisotropy measure *A* ^*eq*^, anisotropy in compressibility *A* ^*B*^, and anisotropy in shear *A* ^*G*^ (or *A* ^*C*^) can all be determined using the standard equations for any symmetry.[^52^, ^53^, ^54^]

$$A^{U}=\;\frac{B_{V}}{B_{R}}+5\frac{G_{V}}{B_{R}}-6\;\geq 0$$

$$A^{B}=\;\frac{B_{V}\;-\;B_{R}}{B_{V\;}+\;B_{R}}$$

$$A^{eq}=\left(1+\;\frac{5}{12}A^{U}\right)+\;\sqrt{{\left(1+\;\frac{5}{12}A^{U}\right)}^{2}-1}$$

$$A_{G}=\;\frac{G_{V}-G_{R}}{G_{V}+G_{R}}$$

Because of its apparent simplicity, the universal anisotropy factor is a preference. The universal anisotropy index *A* ^*U*^, which provides a single anisotropy measurement that is independent of crystal regularity, was suggested by Ranganathan.^[48]^ As we see from above mentioned equations, *G_V_/G_R_* has a significant influence on $A^{U}$ than *B_V_/B*_*R*._ The universal anisotropy factor can only take on positive or zero values. Any deviation from this threshold indicates the presence and degree of anisotropy, and an isotropic crystal is one in which the result is zero. RbV_3_Sb_5_ has highest universal and equivalent Zener anisotropic value at 20 GPa. Extremely elastic behavior can be observed in materials with a high degree of elastic anisotropy. The values of *A* ^*eq*^ clearly predicts the anisotropic behavior of RbV_3_Sb_5_. For an isotropic crystal, *A* ^*G*^ and *A* ^*B*^ are both set to zero. Any value other than zero (positive) indicates a higher anisotropy. According to Table 8, the shear anisotropy is greater than the compressibility anisotropy for RbV_3_Sb_5_, as the *A* ^*G*^ value is greater than the *A* ^*B*^ value.

Perhaps the most comprehensive definition of anisotropy is the universal log‐Euclidean index (*A* ^*L*^). According to,^[46]^ *A* ^*L*^is defined as follows:

$$A^{L}=\sqrt{{\left[ln\left(\frac{B^{V}}{B^{R}}\right)\right]}^{2}+5{\left[ln\left(\frac{C_{44}^{V}}{C_{44}^{R}}\right)\right]}^{2}}$$

In this context, the Voigt and Reuss values of *C_44_* are denoted by $C_{44}^{V}\;$ and $C_{44}^{R}$ , respectively.

$$C_{44}^{R}=\frac{5}{3}\left\{\frac{C_{44}\left(C_{11}-C_{12}\right)}{3\left(C_{11}-C_{12}\right)+4C_{44}}\right\}$$

$$C_{44}^{V}=C_{44}^{R}+\frac{3}{5}\left\{\frac{{\left(C_{11}-C_{12}-2C_{44}\right)}^{2}}{3\left(C_{11}-C_{12}\right)+4C_{44}}\right\}$$

According to,^[46]^ at least 90 % of anisotropic materials have an *A* ^*L*^ value below 1. When it comes to isotropic crystals, *A* ^*L*^ is zero, but as the anisotropy rises, it grows. The *A* ^*L*^ values of RbV_3_Sb_5_ crosses the anisotropic conditions. Moreover, at GPa, where it crosses the usual limit, so thus the degree of anisotropy increases. It is worth mentioning that materials with higher *A* ^*L*^ possess layered structural features and vice versa.

The evaluation of the linear compressibility's of RbV_3_Sb_5_ along the a‐ and c‐axis ($\beta_{a}$ and${\;\beta }_{C}$ ) was based on the equation in^[55]^:

$$\beta_{a}=\frac{C_{33}-C_{13}}{D}\;\mathrm{AND}\;\beta_{C}=\frac{C_{11}+C_{12}-{2C}_{13}}{D}$$

$$\mathrm{With}\;D=\left(C_{11}+C_{12}\right)C_{13}-2{\left(C_{13}\right)}^{2}$$

In the case of isotropic compressibility, the ratio of the coefficients ($\beta_{c}$ /$\beta_{a}$ ) is one, whereas any discrepancy highlights the anisotropy in the compressibility. Anisotropy is quite noticeable in the predicted values.

**Figure 4:**
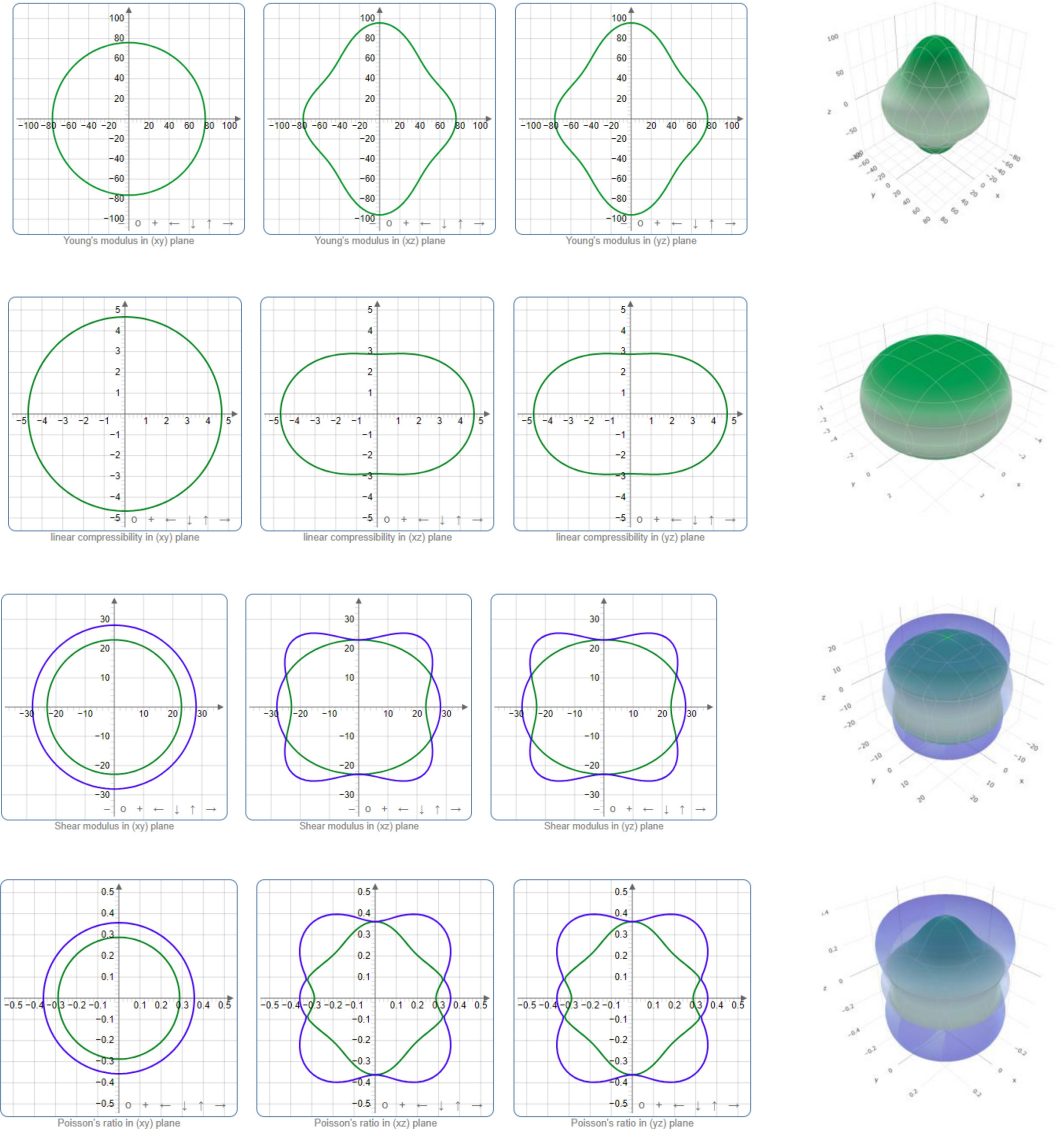
Directional dependency of several parameters (Young's modulus, linear compressibility, shear modulus, and Poisson ratio).

An analysis was conducted to determine the directionality of the Young's modulus, linear compressibility, shear modulus, and Poisson ratio. Graphical representations in both two and three dimensions are part of this approach. The compound's 2D projection on the xy‐, xz‐, and yz‐planes, along with the 3D image of Young's modulus, linear compressibility, shear modulus, and Poisson ratio are shown in Figure 4. Graphical depictions of uniformly shaped 2D and 3D crystals reflect their isotropic nature. The larger the deviation from these ideal shapes, the larger the anisotropy. Parameter minimum and maximum values are shown by the green and blue curves, respectively. In the xy‐plane, all four parameters are isotropic, while in other planes, they are anisotropic.

### 2.4 Acoustic and Thermodynamic Properties

Several acoustic properties were investigated in this study, including acoustic impedance, the speed of sound and its anisotropy, and the sound radiation coefficient. The second‐order elastic constants provide insights into the propagation characteristics of acoustic waves within a given material.

The parameters that reveal the thermal behavior of a material, including the Debye temperature, melting temperature, thermal conductivity, and thermal expansion coefficient, are investigated in order to forecast the system's potential applications. These properties were investigated at various hydrostatic pressures. A comprehensive summary of all the computed thermo‐physical and acoustic properties of RbV_3_Sb_5_ is presented in Table 9. A strong correlation exists among the mechanical stability, phonon dispersion, thermodynamic property, and superconducting property. Additionally, a correlation existed between this and the Debye temperature. The equation representing the Debye temperature at T=0 K is as follows:,^[56]^

$$\theta_{D}=\frac{h}{k_{B}}{\left(\frac{3n\rho N_{A}}{4\pi M}\right)}^{\frac{1}{3}}V_{m}$$

**Table 9:** The calculated values of Debye Temperature (θ_D_) and thermal expansion coefficient of RbV_3_Sb_5_ at different hydrostatic pressures (GPa).

P (GPa)	Density ρ	V_T_	V_L_	V_A_	Z(X10^6^)	I	Debye Temperature θ_D_ (K)	*T_m_* (K)	*α* (X10^−5^) (K^−1^)
0	4194.14	2532.95	5310.00	2848.87	10.62	0.60	284.39	913.29	5.91
5	4896.18	2816.15	5539.11	2648.24	13.78	0.575	278.11	1100.85	4.12
10	5197.74	2724.77	5835.077	2540.83	14.16	0.524	272.356	1274.25	4.15
15	5467.03	2892.49	5756.406	2698.22	15.81	0.529	294.02	1282.845	3.49
20	5705.41	3006.17	6605.163	2832.78	17.15	0.527	312.91	1181.49	3.10
22	5776.52	3128.84	6285.418	2916.86	18.07	0.542	323.57	1536.66	2.83

V_m_ represents the mean speed of sound, which is determined by both the transverse sound wave velocity (V_T_) and the longitudinal sound wave velocity (V_L_). Additionally, k_B_ denotes the Boltzmann constant, h signifies Planck's constant, and n symbolizes the quantity of atoms per molecule. Furthermore, ρ, M, and N_A_ represent the parameters of density, molecular weight, and Avogadro's number, respectively. The transverse (shear), longitudinal, and average sound velocities in a crystalline material are determined by the following equations, respectively^[50]^:

$$V_{m}={\left[\frac{1}{3}\left(2V_{T}^{-3}+V_{L}^{-3}\right)\right]}^{-\frac{1}{3}}\mathrm{where},\;$$

$$V_{T}=\sqrt{\frac{G}{p}}\;\mathrm{and}\;V_{L}=\sqrt{\frac{3B+4G}{3P}}$$

Greater Debye temperatures correspond to increased phonon thermal conductivity in crystals. The Debye temperature of materials differentiates the classical and quantum‐mechanical characteristics of phonons. When the thermal energy of a system exceeds its Debye temperature, it results in the excitation of all the phonon modes. The Debye energy serves as the maximum energy limit for the phonon modes. At temperatures over θ_D_, it is anticipated that all modes of vibrations for a solid will possess equivalent energy levels. Acoustic and optical phonon modes are expected to dominate the lattice below and above θ_D_, respectively. Electron‐phonon interaction is anticipated to be weak for temperatures below θ_D_. Due to the temperature‐dependent increase in electron mobility, the interaction between optical modes and electrons in solids is more pronounced compared to acoustic modes. The compound's Debye temperature, determined at 0 K and 0 GPa, is 284.39 K (Table 9). The Debye temperatures corresponding to various hydrostatic pressures are also shown in Table 9. The Debye temperature exhibits a slight fluctuation as pressure increases. Debye temperatures exhibit higher values for materials with greater hardness, such as diamond, and lower values for materials with lesser hardness, such as lead. The computed Debye temperatures of the structure imply that it is a soft material. The speed of sound in a metal is exactly proportional to its Debye temperature in acoustic tests.

The acoustic velocities and acoustic impedances Z estimated at various pressures are presented in Table 9. The relationship between velocities and pressure exhibits a nonmonotonic pattern. Efforts have been made to categorize materials based on acoustic signals to enhance the capabilities of humanoid robots.^[51]^ Based on the sound velocity classification,[^57^, ^58^] our material exhibits a low velocity. Acoustic impedance (Z) is a commonly utilized parameter in various scientific fields. It quantifies the extent of acoustic energy transfer between two distinct materials. The velocity of sound is greater in materials with higher Z. The disparity in acoustic impedance between the interfaces of the two medium finds extensive use in various fields such as the music industry, medical ultrasound imaging, acoustic sensors, aerospace industry, transducer design, industrial factories, autos, and underwater acoustic applications. The expression provided represents the acoustic impedance of a solid.^[37]^:

$$Z=\sqrt{\rho G}$$

Where, *ρ* and *G* are the density and shear modulus of the medium. At higher temperatures, the acoustic impedance of denser and stiffer materials is greater. The quantity of energy reflected and transmitted when a sound wave arrives at an interface is explained by the degree to which the acoustic impedance of the two mediums is misaligned. The reflection coefficient (R) is a measure of the amount of sound that is reflected at an interface; acoustic impedance studies of materials are crucial for this estimation.^[59]^

The acoustic radiation coefficient (I) quantifies the degree to which the vibration of the specimen is attenuated as a result of acoustic radiation. The acoustic radiation coefficient of a material can be determined using the following formula:

$$I=\sqrt{\frac{G}{\rho^{3}}}$$

This characteristic is essential for selecting appropriate materials for constructing musical instruments with high and low pitches. The calculated acoustic radiation coefficient is listed in Table 9. The acoustic conversion efficiency (ACE) of a medium is directly proportional to its acoustic radiation coefficient.

The melting temperature (T_m_) is crucial in determining suitable materials for thermal management in industrial applications. Low and high T_m_ materials are suitable options for thermal interface material (TIM) and thermal barrier coatings, respectively. The melting temperature is directly influenced by factors such as the strength of bonding, more cohesive energy, and a reduced coefficient of thermal expansion.^[58]^ The T_m_ of the material was determined using the following equation.^[60]^


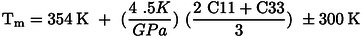




The ground state of RbV_3_Sb_5_ has an estimated value of T_m_ at 913.29 K, as seen in Table 9. The pressure dependency of a substance's melting temperature is widely recognized. Consequently, we are also investigating the relationship between pressure and the melting temperature of our material. Table 9 demonstrates that the melting temperature for RbV_3_Sb_5_ rises as the pressure increases, however the change is not consistently in one direction. The phenomenon of an increase in both the melting temperature and Debye temperature with pressure is widely seen.

The thermal expansion coefficient (TEC) is a fundamental thermal attribute of materials that is related to the regular vibrations of the lattice structure. The calculated values of α at 300 K at different pressuresare given in Table 9. The material's properties that can be measured include thermal conductivity, specific heat, entropy, and isothermal compressibility. Low thermal expansion materials are highly sought after in the ceramic sector due to their extensive applicability in areas such as high anti‐thermal shock applications, electronic devices, heat‐engine components, and spintronics devices.^[52]^ TEC of a material can be determined by utilizing the shear modulus through the following equation^[58]^:

$$\alpha =\frac{1.6\times 10^{-3}}{G}$$

### 2.5 Phonon Property and Molecular Dynamics

**Figure 5:**
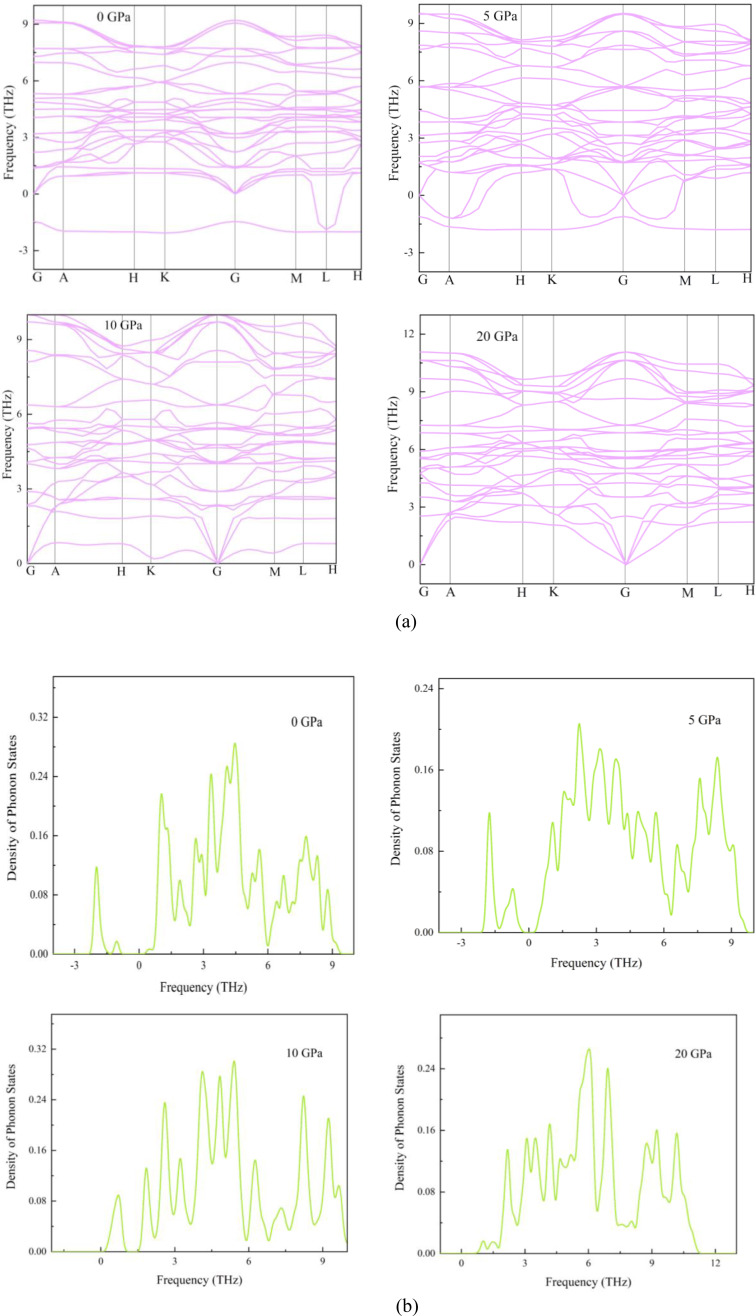
(a) Phonon dispersion curves and (b) phonon density of states of structure under different hydrostatic pressure.

An analysis of phonon dispersion curves and phonon density of states can offer useful insights into the structural stability and vibrational characteristics of materials.[^61^, ^62^] Moreover, these methods enable the analysis of thermodynamic properties such as thermal expansion, heat capacity, and Helmholtz‐free energy. DFT technique was utilized to produce the phonon dispersion curves for various levels of applied pressure on the RbV_3_Sb_5_ structure. Figure 5 depicts these curves along the main axis of symmetry in the Brillouin zone (BZ), namely from point G to point A to point H to point K to point G to point M to point L to point H. Furthermore, Figure 5 provides the phonon density of states for all configurations. Based on the findings, the pristine structure has mechanical stability, however it is thermodynamically unstable. The immaculate structure displays negative frequency values when measured in the terahertz (THz) unit. The frequency described above is widely known as the imaginary phonon frequency. Dispersion curves with negative frequency values suggest structural dynamical instability in the ground state. The final outcome of instability is the disturbance of crystal symmetry. Phonon computations were also performed on structures subjected to stress. At hydrostatic pressure of 5 GPa, the structure becomes unstable due to the presence of negative frequency values. Under mounting pressure, the structure achieved stability. There is no phonon branch with negative energy in the structure under a stress of 10 GPa. This indicates that the Kagome compound is stable under high pressure and temperature conditions.

**Figure 6:**
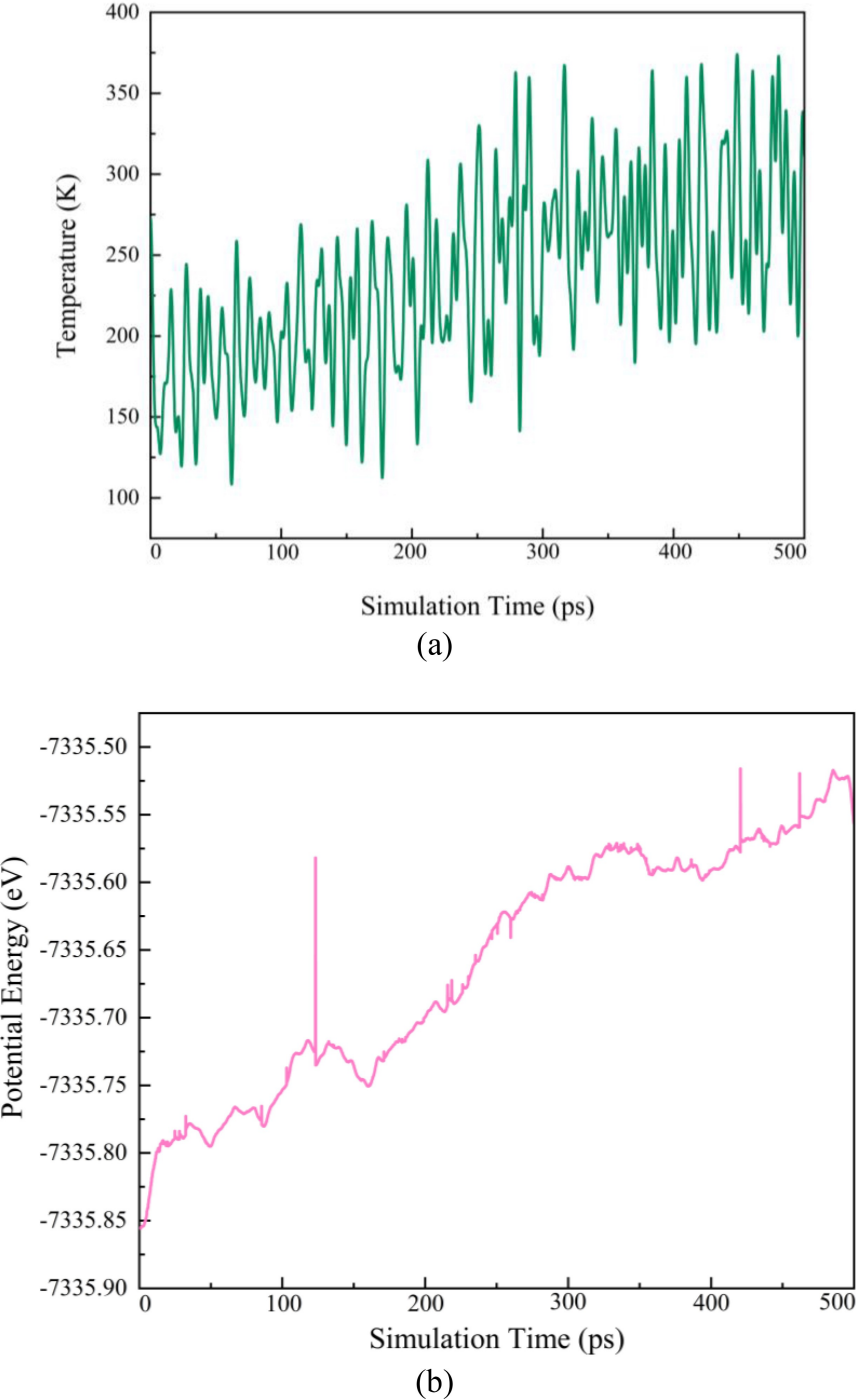
(a) Temperature profiles and (b) Potential energy of structure (simulation time up to 500 ps) in the equilibrium condition.

In MD simulations, the system's energy and temperature play a crucial role in determining its thermodynamic state. When temperature and energy fluctuations range around 5 % and 10 %, the structure is considered to be in equilibrium. Figure 6 depict the potential energy and temperature profiles of the simulated systems during the 500 ps long equilibrium trajectory. Neither potential energy nor temperature remained in a relatively constant range, which indicates thermodynamic instability in the pristine structure.

### 2.6 Fermi Surface Topology

**Figure 7:**
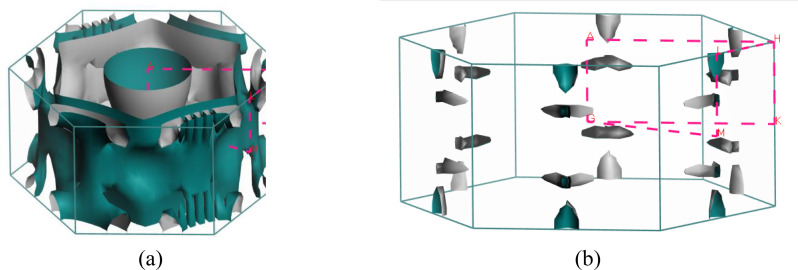
The Fermi surfaces of RbV_3_Sb_5_ for band (a) 37 and (b) 38 at 5 GPa.

**Figure 8:**
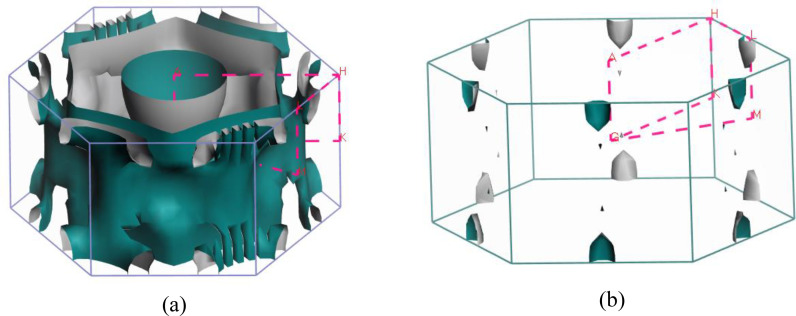
Fermi surfaces of RbV_3_Sb_5_ for band (a) 37 and (b) 38 at 5 GPa.

**Figure 9:**
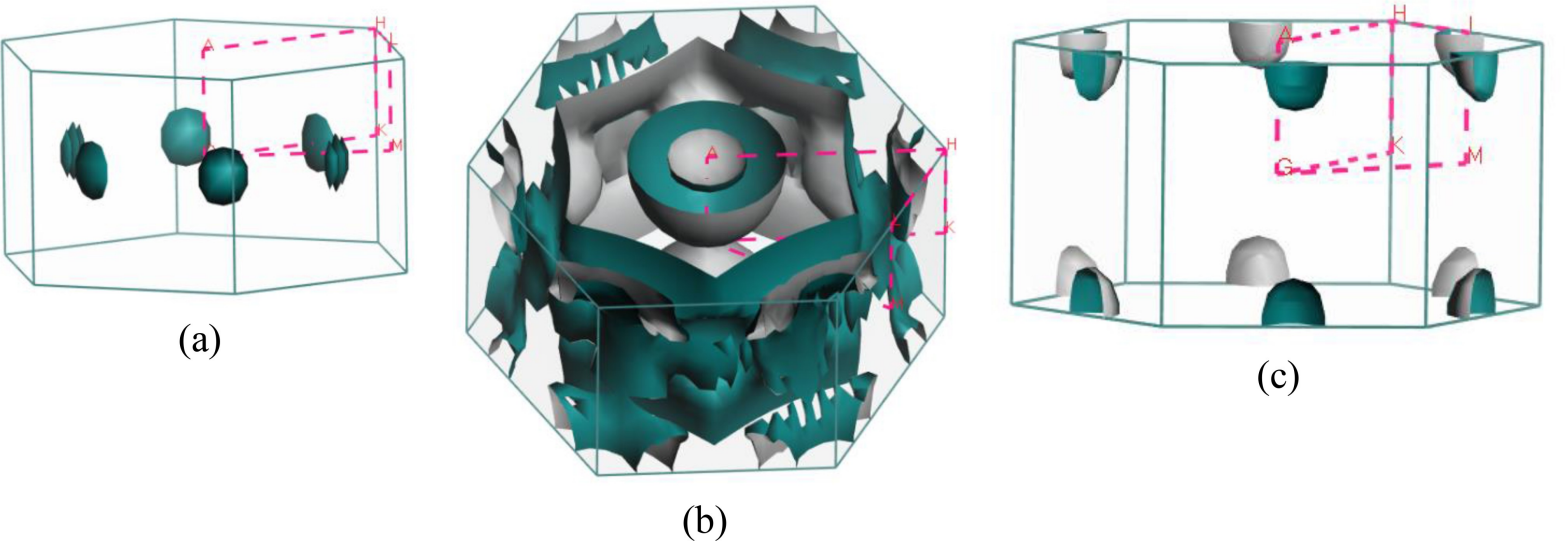
Fermi surfaces of RbV_3_Sb_5_ for band (a) 36 and (b) 37 (c) 38 at 10 GPa.

**Figure 10:**
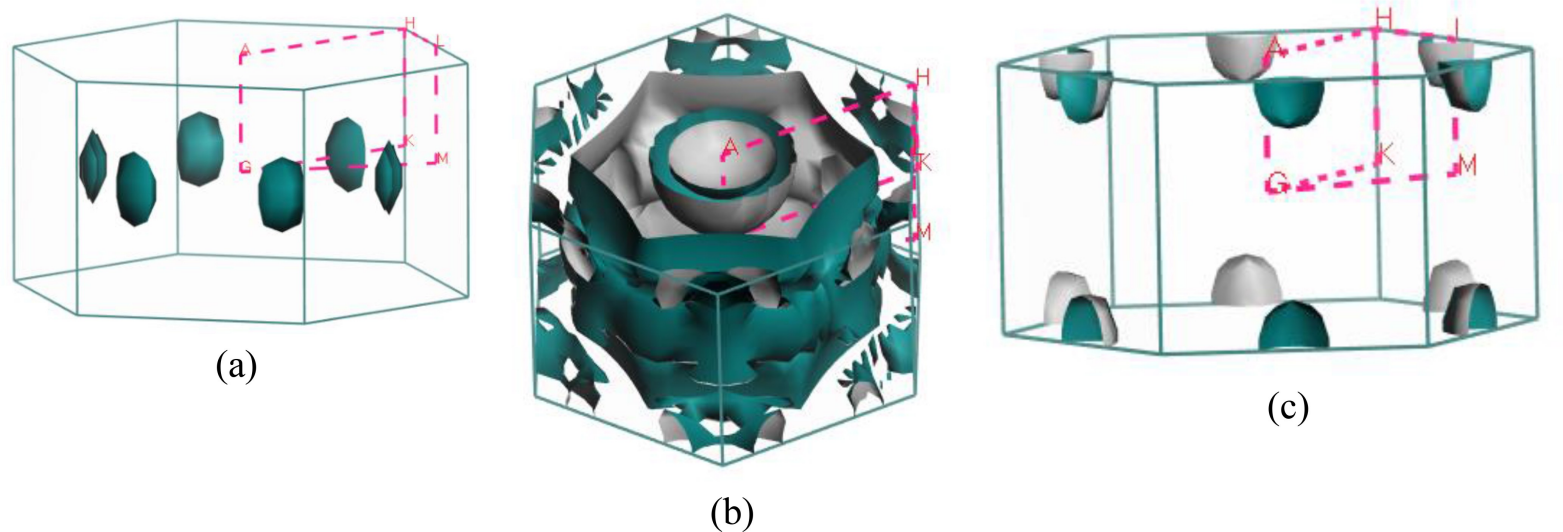
Fermi surfaces of RbV_3_Sb_5_ for band (a) 36 and (b) 37 (c) 38 at 15 GPa.

**Figure 11:**
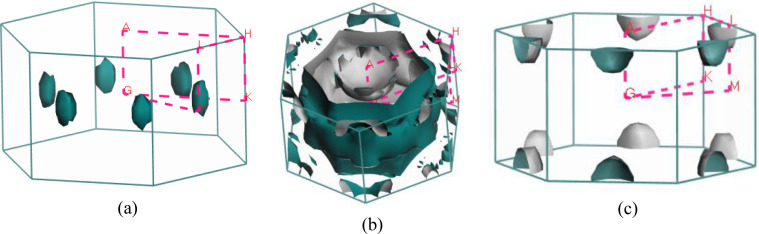
Fermi surfaces of RbV_3_Sb_5_ for band (a) 36 and (b) 37 (c) 38 at 20 GPa.

**Figure 12:**
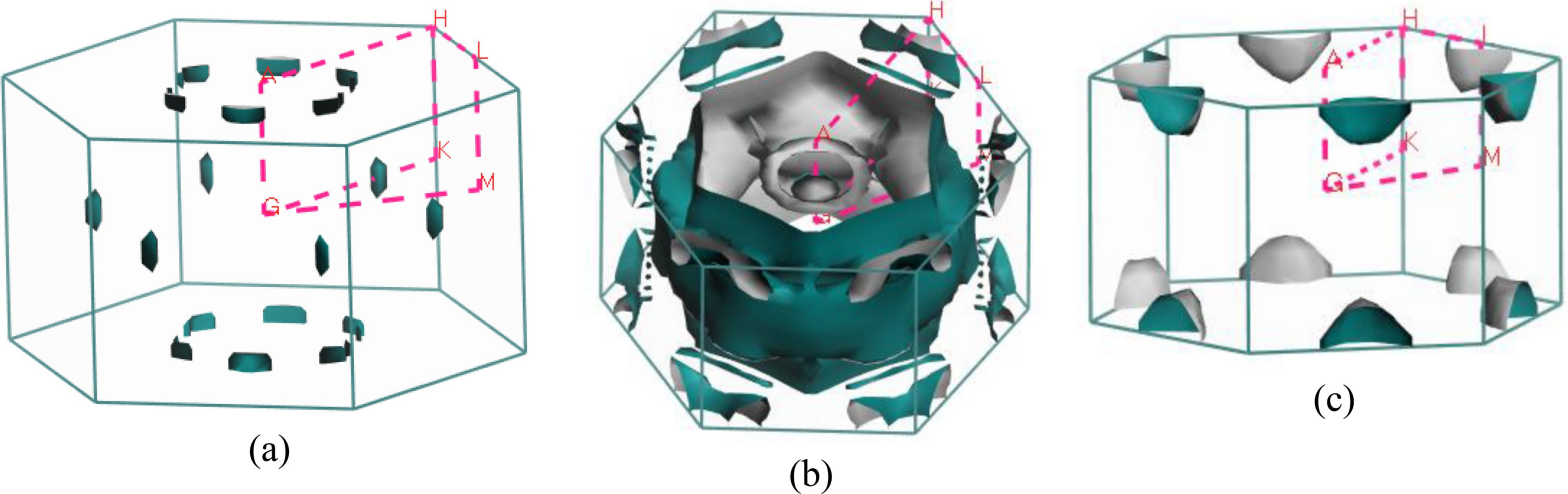
Fermi surfaces of RbV_3_Sb_5_ for band (a) 36 and (b) 37 (c) 38 at 22 GPa.

Predicting several properties like electrical, magnetic, optical, and thermal properties of metals and semimetals is made easier with an understanding of the Fermi surface (FS). The development of the superconducting state involves electrons close to the Fermi sheets.^[34]^ Figures 7, 8, 9, 10, 11, 12 show the Fermi surface of the RbV_3_Sb_5_ Kagome compound at various pressures. Electron‐ and hole‐like sheets are present in the topology. In the figures, the dotted line represents points of high symmetry. In the ground state, we obtained two bands (37 and 38) which contributes in fermi surface. The electrical characteristics are strongly two‐dimensionally implied by a cylinder‐like ground state field structure that is centered on the Gamma‐point (or along the G−A path) for band 37. The Gamma‐point is also the center of a big and complicated electron or hole‐like hexagonal sheet which are almost parallel for all pressure. For band 38, there is a little sheet that resembles an electron and a tiny hole, respectively. The structure of the Fermi surface changes dramatically as the pressure increases. Band 38′s contribution to the total Fermi surface grows with increasing pressure. Electronic pockets of hexagonal symmetry are generated by band 38. An further band, band 36, adds to the Fermi surface for pressures greater than 10 GPa, and it crosses the Fermi level. The result is a complicated structure consisting of six separate Fermi sheets (Figures 7, 8, 9, 10, 11, 12).

### 2.7 Electronic Property

Many microscopic phenomena, including chemical bonding, electronic transport, superconductivity, optical response, and magnetic order, can only be explained by studying a material's electronic band structure. The electronic properties are mostly influenced by bands in close proximity to the Fermi level. In order to faithfully depict nanostructures and electrical devices, it is vital to possess exact band topologies and authentic effective masses of charge carriers. Studying the electronic band structures of materials is essential for catalyst design,^[63]^ the speedy identification of “battery materials”,^[64]^ and the development of superconducting materials.^[26]^ The flat bands found in various Kagome materials originate from the orbital interactions of the Kagome lattices. The existence of both flat and Dirac bands around the Fermi level in structures can give rise to superconducting potential. The exceptional mobility is a consequence of the minuscule effective masses of the charge carriers combined with the corresponding wave vectors, which are determined by the quasi‐linear energy dispersion. This highlights the need of investigating the band structure of the chemical.

**Figure 13:**
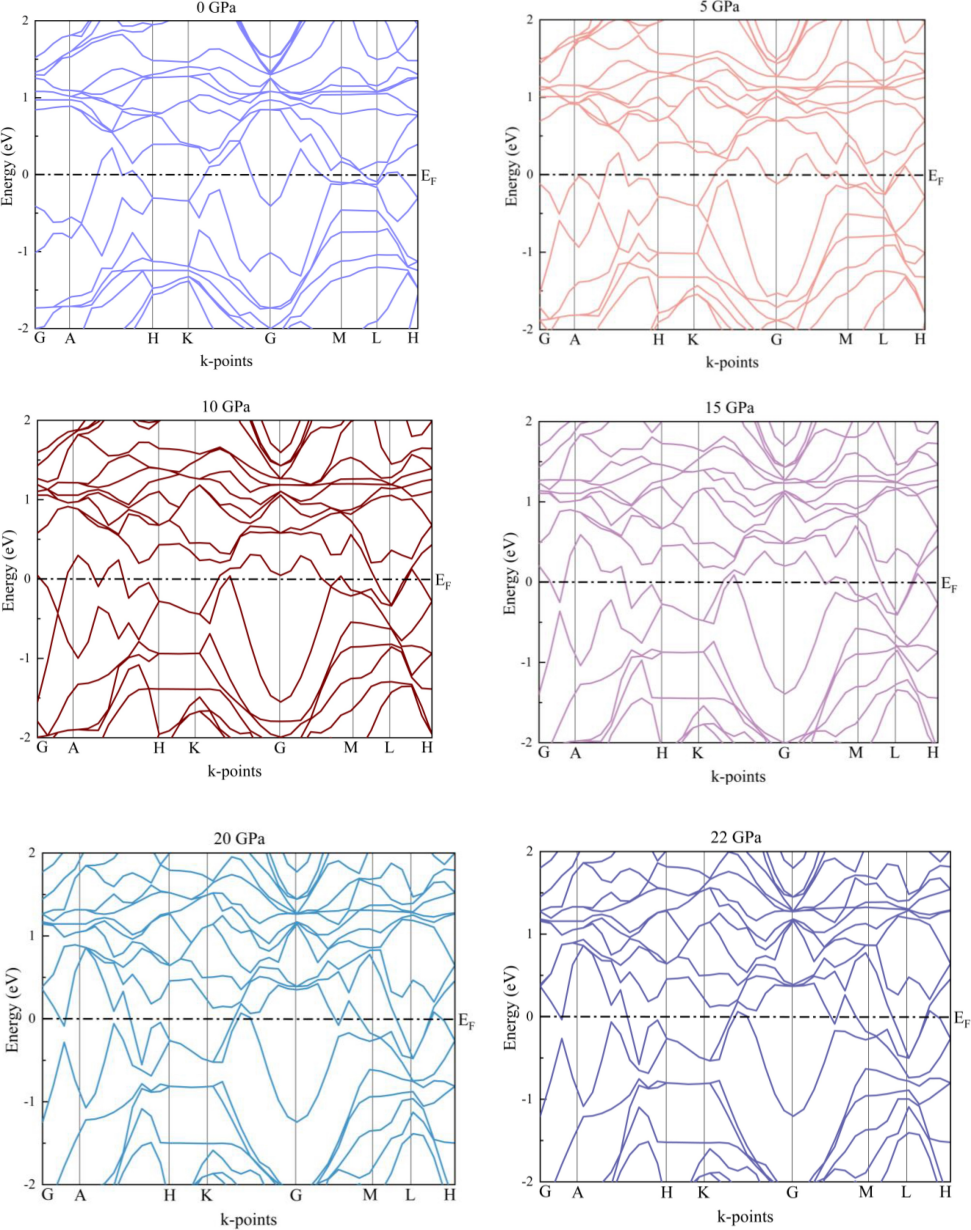
Band structures of the RbV_3_Sb_5_ structure under several hydrostatic pressures.

By analyzing band structures and Partial Density of States (PDOS), we probed electronic characteristics. For this reason, we have investigated the Kagome material's band topology by means of its electrical band structure. We estimated the band structures along the symmetry points of the Brillouin zone, which are G→A→H→K→G→M→L→H. At different hydrostatic pressures, the electronic energy band structures of RbV_3_Sb_5_, computed on a discrete k‐mesh along the high symmetry directions in the first Brillouin zone, are displayed in Figure 13, using a smearing width of 0.1 eV. The presence of a distinct overlap between the conduction and valence bands at the Fermi level provides evidence of the metallic character. In the ground state, the bands that cross the Fermi level are shown by band numbers 37 and 38, respectively. Several places in the Brillouin zone exhibit degeneracy of these two bands due to excessive symmetry. In some regions of the E(k) diagram, one can see dispersive bands that are nearly linear. A pressure of 10 GPa causes three bands to intersect at the Fermi level. In certain regions of the BZ, one can observe characteristics similar to electrons and those similar to holes.

**Figure 14:**
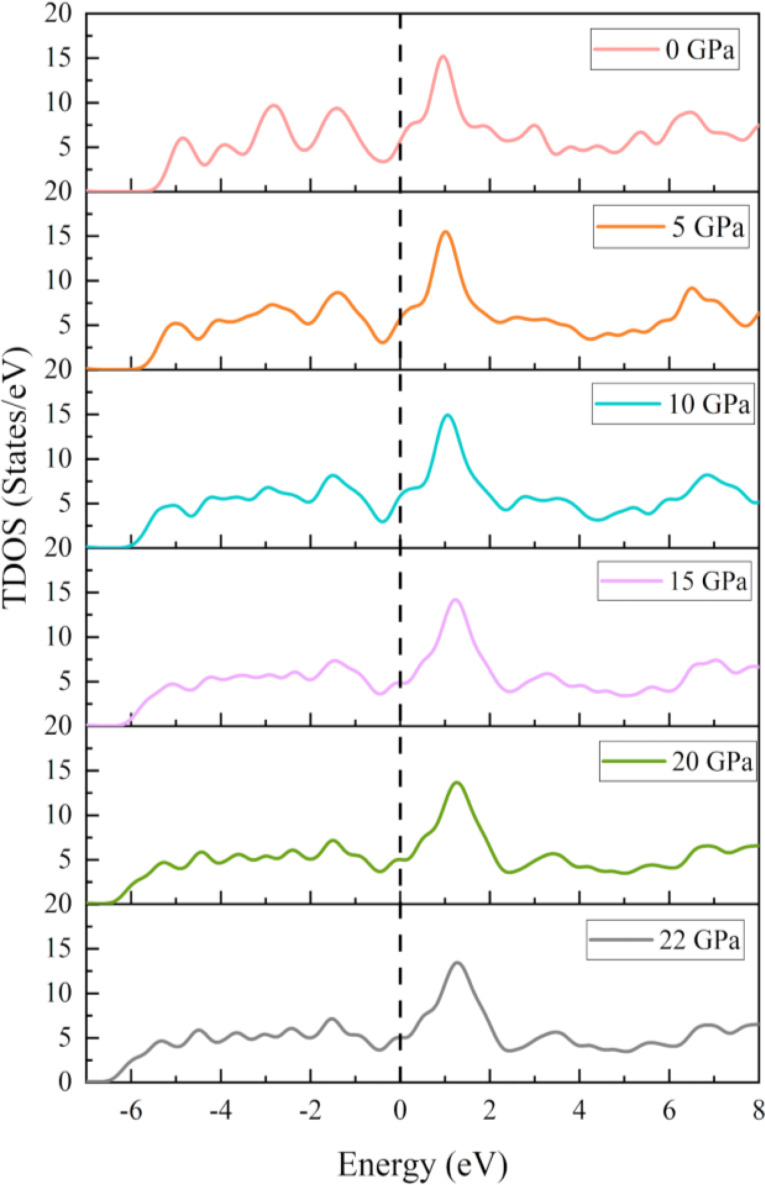
TDOS of the RbV_3_Sb_5_ structure under several hydrostatic pressures.

**Figure 15:**
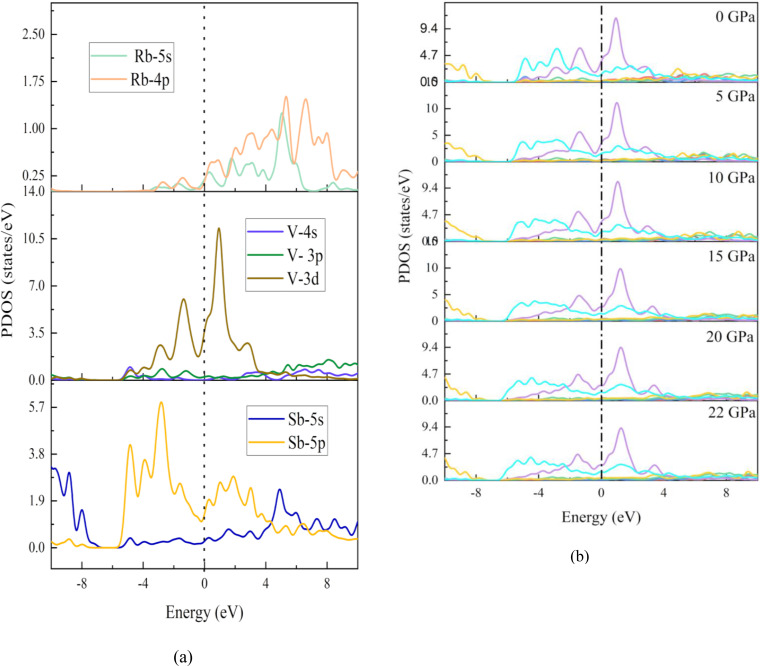
(a) PDOS of the pristine RbV_3_Sb_5_ structure and (b) comparison of PDOS of RbV_3_Sb_5_ structure under several hydrostatic pressures.

The spin‐polarized PDOS was calculated and we obtained identical graphs for both spins. Since the structure is non magnetic, spin polarized DOS is not applicable here. To understand the impact of atoms and orbital's on a material's thermal, optical, and electrical conductivity, as well as its other electronic transport properties, it is advisable to examine its total DOS and PDOS. Also, it is closely related to the electronic density of states at the Fermi level, N(E_F_),[^65^, ^66^] and it controls a number of important physical variables, such as the electronic contribution to metal heat capacities and spin paramagnetic susceptibilities. At various hydrostatic pressures, the total and partial DOS of RbV_3_Sb_5_ are displayed in Figures 14 and 15. At zero energy, the vertical broken line represents the Fermi level. This chemical is metallic because its total PDOS is finite at the Fermi level.

The PDOS graphics clearly show the Sb‐5p electronic states are the primary contributors to the low energy valence bands ranging from −6 eV–−2 eV. The bands at the Fermi level are primarily caused by the strongly hybridized V‐3d and Sb‐5p electronic states, in which Rb atoms also play a role (Figure 14). Hybridization around the Fermi energy point is a good sign that strong covalent bonds have formed. We anticipate that, among all atomic orbitals, the three‐dimensional electrons of V atoms will have the greatest impact on electronic characteristics, since their three‐dimensional states predominate in the PDOS around the Fermi level. The overall PDOS in the (2 eV–8 eV) conduction band, however, is derived from the hybridization of the Cs‐5p and 6 s states and the Sb‐5p and 5 s levels. Most noticeably affected by variations in pressure are the atomic resolved PDOS of the V atom.

**Figure 16:**
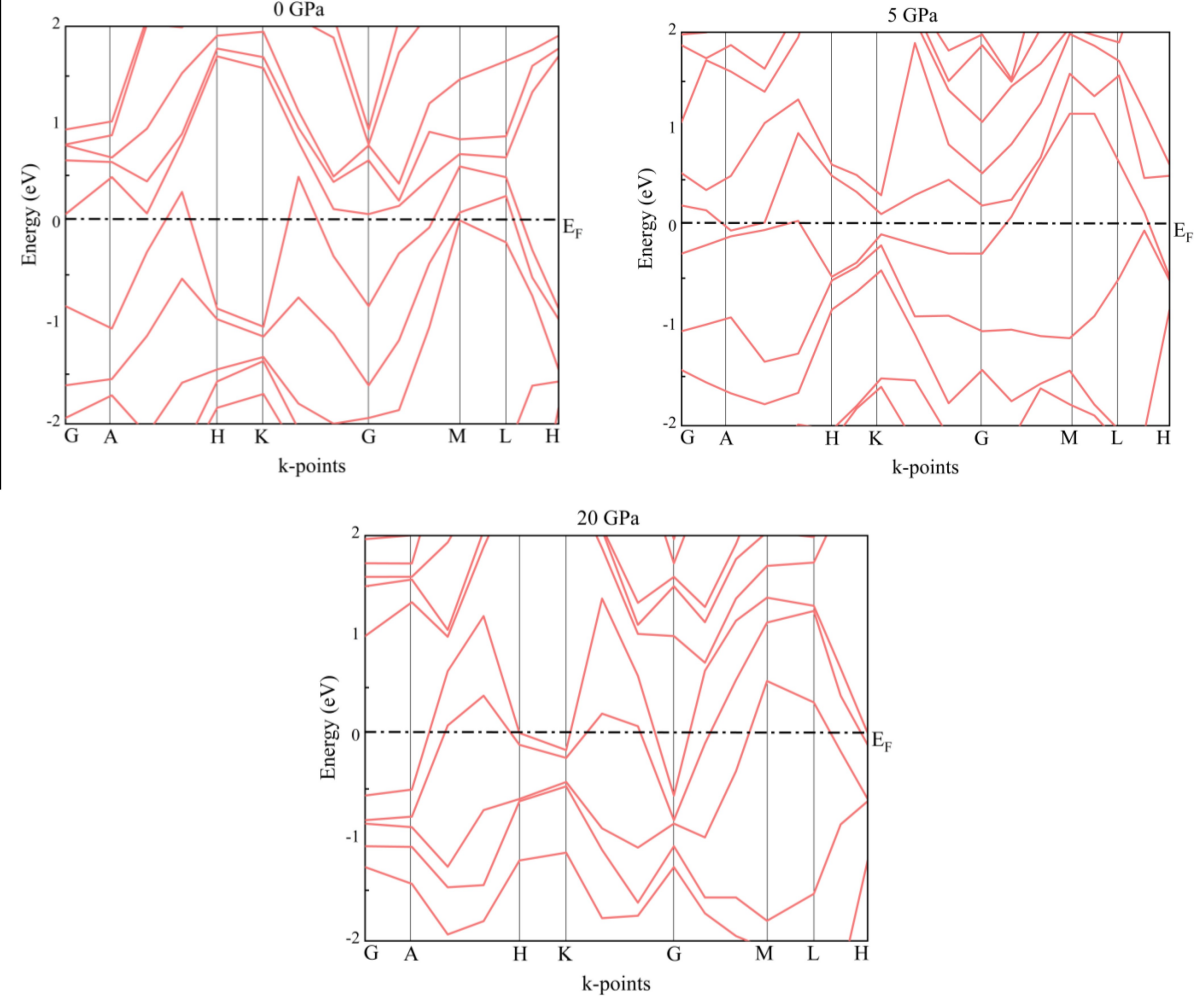
Band structures of the RbV_3_Sb_5_ structure under several hydrostatic pressures using HSE06 hybrid functional.

We also estimated the band structures of substances using the HSE06 hybrid exchange function. This is because a different approach that is based on a precise handling of the partial exchange energy is applied in this case. It is better to convert only a piece of the non‐local exact exchange to a (semi)‐local exchange expression. For this reason, HSE06 offers a more accurate estimate of the solids’ ground state. The Figure 16 indicates the band structure using the hybrid functional. We analyze that there are similarities between GGA‐PBEsol calculations and HSE06 calculations. For both exchange functionals RbV_3_Sb_5_ shows metallicity in pristine as well as stressed conditions.

In the ground state, the computed total PDOS of RbV_3_Sb_5_ at E_F_ is 5.71 states per eV per unit cell, which is in line with previous research.^[28]^ When figuring out electronic correlations in a system, the repulsive Coulomb pseudopotential, another name for the electron‐electron interaction (EEI) caused by the Coulomb force, which is a key factor.

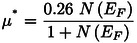




Strong electronic correlations are indicated by the high calculated Coulomb pseudopotential of 0.22 for RbV_3_Sb_5_ in the ground state. By decreasing the effective electron‐phonon coupling constant, the repulsive Coulomb pseudopotential prevents the production of Cooper pairs in superconductors.[^67^, ^68^] Table 10, which is located below, displays the values of N(E_F_) and μ* as they vary on pressure. As seen in Table 10, the TDOS at the Fermi level and Coulomb pseudopotential exhibit weak pressure dependence.

**Table 10:** The TDOS at the Fermi level, N(E_F_) (states/eV.unit cell) and Coulomb pseudopotential, μ* of RbV_3_Sb_5_ under different hydrostatic pressures (GPa).

P	N(E_F_)	*μ* ^*^
0	5.71	0.22
5	5.69	0.221
10	5.83	0.222
15	4.84	0.215
20	5.02	0.216
22	5.05	0.217

### 2.8 Optical Properties

Optical parameters can provide valuable insights into various physical aspects. For instance, the reflection of light by substances can reveal information about their electrical states. Throughout our investigation, we calculated the optical characteristics by analyzing electronic transitions. The Kramer‐Kronig dispersion relation is employed to determine the complex component of the dielectric function. Both the actual and imaginary parts of the dielectric function can be employed to estimate optical properties (which vary with energy), such as absorption, reflection, optical conductivity, and dissipation. These specific attributes are closely linked to the refractive index of the material. Electronic transitions arise when materials absorb input photons, causing previously unoccupied states of compounds to become occupied ones. The level of photon attenuation is directly proportional to the number of occupied states. This process leads to a rise in the number of occupied states, resulting in a higher absorption of light photons and an elevation in the dielectric constant. The equation of refractive index and dielectric constant are N=n(w)+ik(w) and ${\;\epsilon =\epsilon }_{1}\left(\omega \right)+{\;\epsilon }_{2}\left(\omega \right)$ .

It was observed that the refractive index is mathematically related to the square root of the dielectric function. Consequently, the graphs representing the real part of refractive index n(w) and real part of dielectric constant ${\;\epsilon }_{1}\left(\omega \right)$ had a strong resemblance to each other. In terms of energy storage capacity, the real part of the dielectric constant${\;\epsilon }_{1}\left(\omega \right)$ is measured, and imaginary part${\;\epsilon }_{2}\left(\omega \right)$ is employed to quantify the light absorption due to neuronal charge excitations within the material.^[69]^

**Figure 17:**
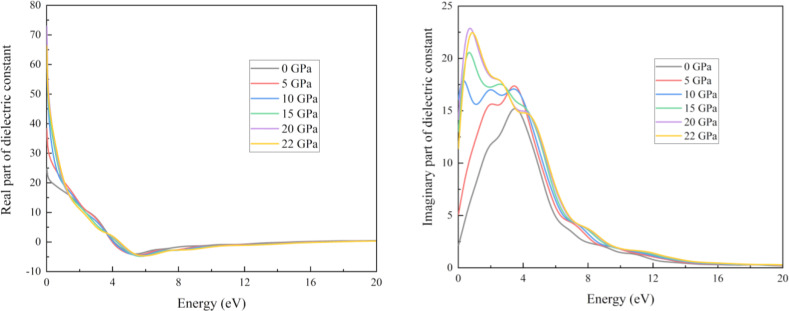
Real and imaginary part of the dielectric function of the RbV_3_Sb_5_ structure under various applied hydrostatic pressures.

**Figure 18:**
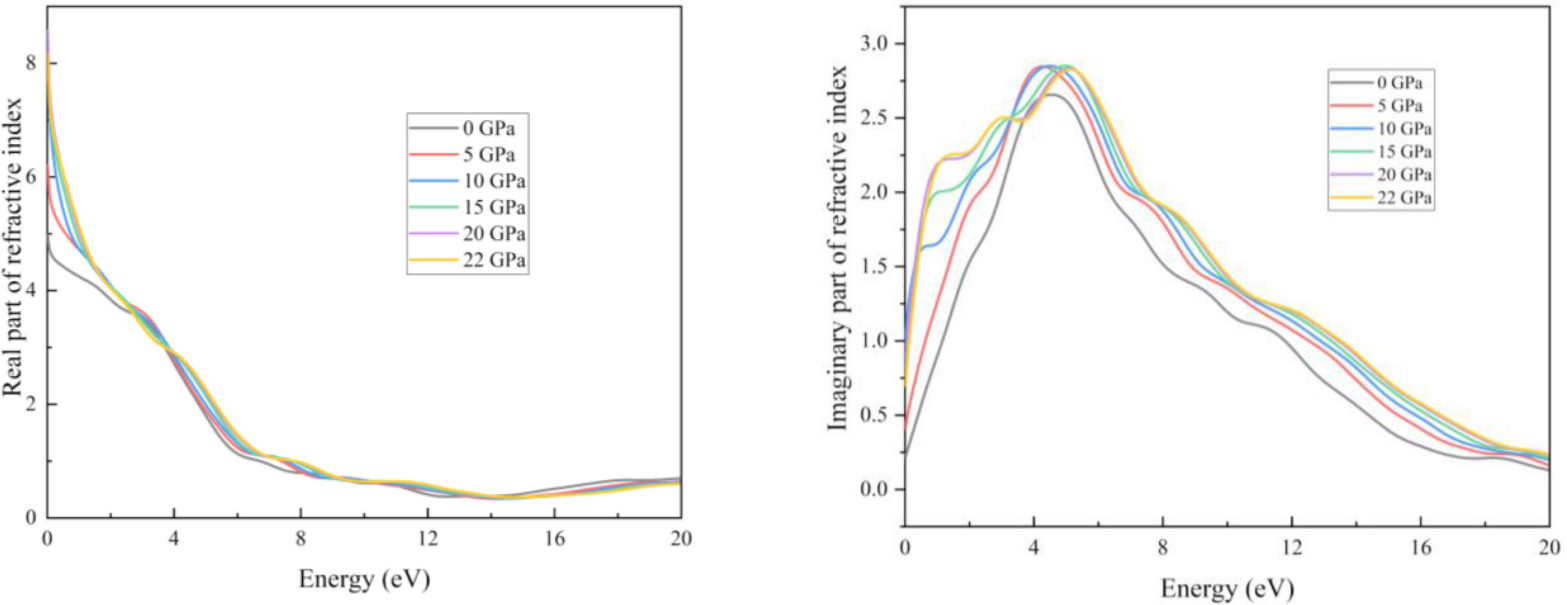
Real and imaginary part of the refractive index of the RbV_3_Sb_5_ structure under various applied hydrostatic pressures.

There is a robust relationship between the imaginary part of a substance and its transparency. The correlation between the refractive index value and ${\;\epsilon }_{1}\left(\omega \right)$ results in a comparable pattern which is observed in Figures 17 and 18. The material's propagation and dissipation capabilities of electromagnetic photon fields are represented by the functions n(ω) and k(ω), respectively. This term is fundamental to the operation of many optical devices, including quantum computers, LEDs, photo‐catalysts, and more.^[15]^ In addition, this statement clarifies the optical dispersion of the composite. There is no extra peak that resembles reflectivity and dielectric constant as well as structure shows a peak for these two characteristics at zero eV energy. The values enclosed by this region decreased as the photon energy increased. The excited states used in our DFT optics research are the unoccupied Kohn‐Sham states. Within the presence of an electric field, photons cause states to change from unoccupied to occupy. These electronic transitions are responsible for the imaginary component of the dielectric function and conductivity.^[70]^

**Figure 19:**
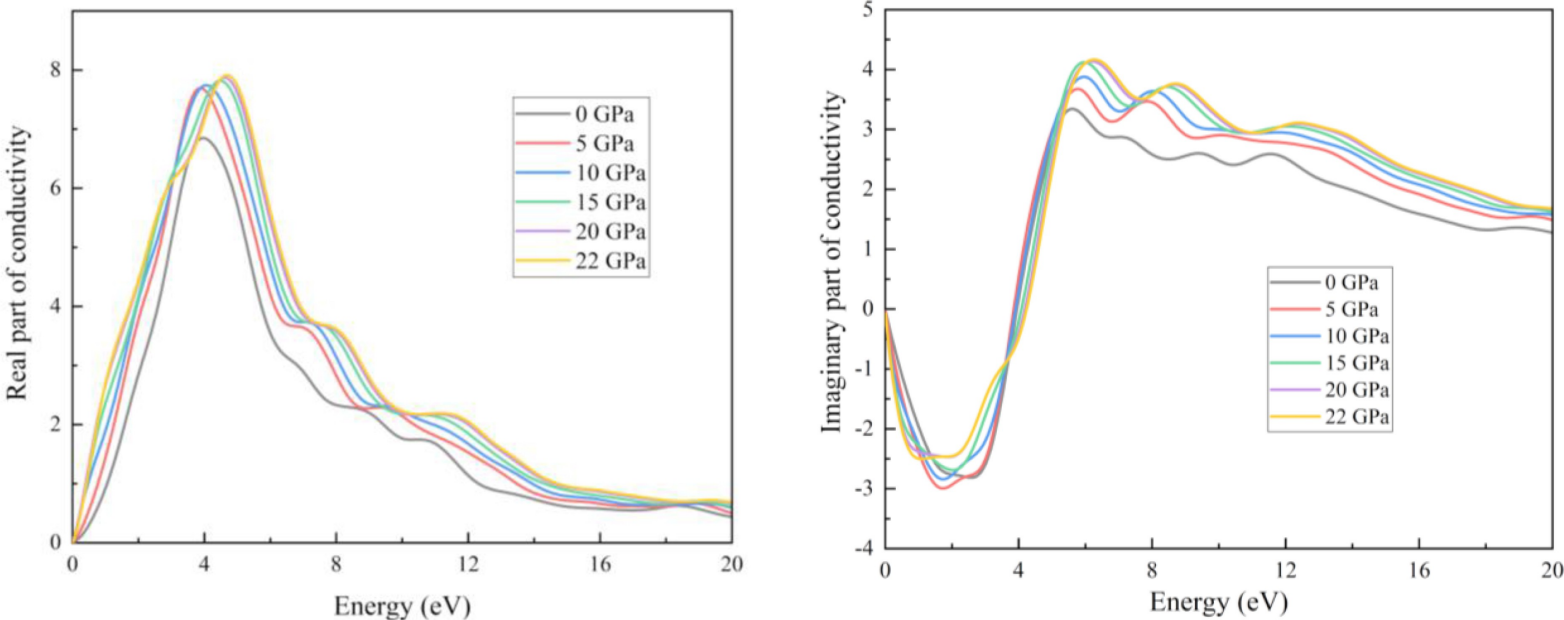
Real and imaginary part of the conductivity of the RbV_3_Sb_5_ structure under various applied hydrostatic pressures.

**Figure 20:**
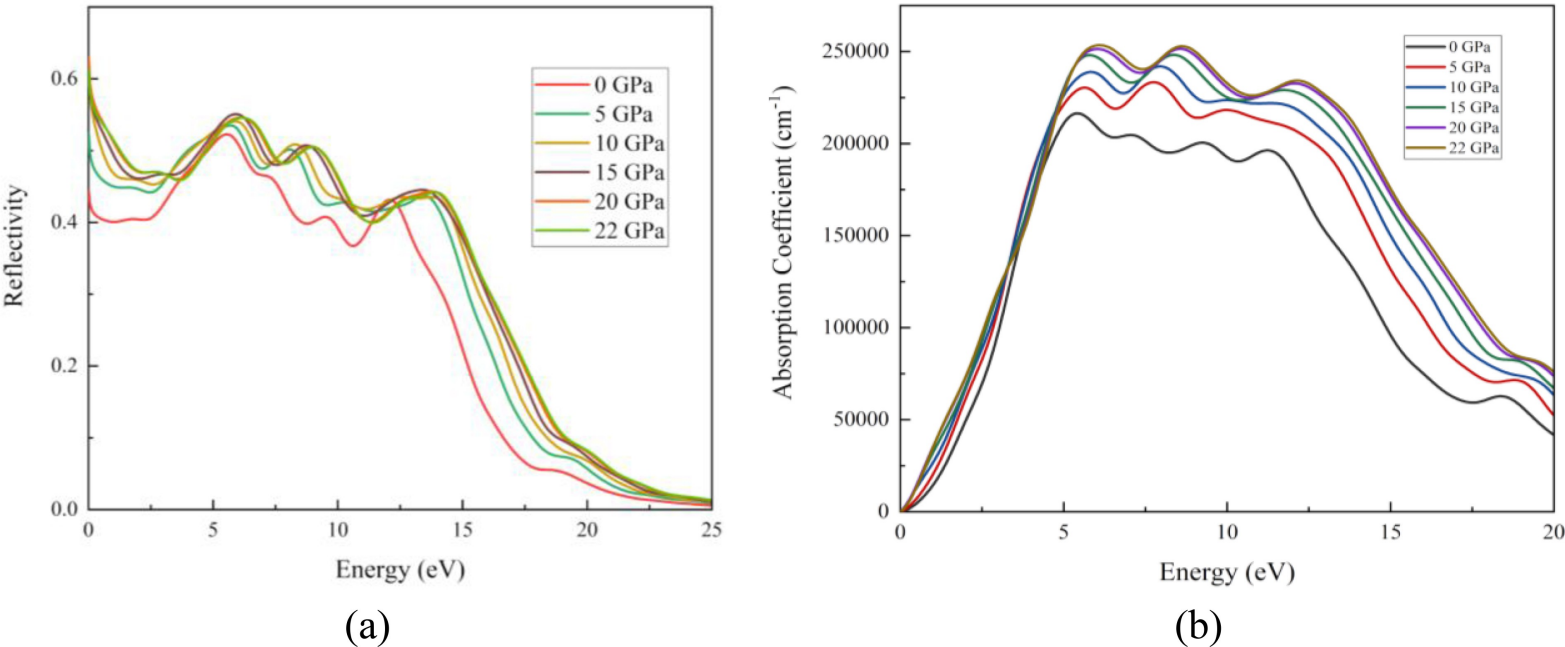
(a) Reflectivity and (b) absorption coefficient of the RbV_3_Sb_5_ structure under various applied hydrostatic pressures.

Figure 19 represents the graphs for reflectivity and absorption coefficient. For pristine structure, the maximum photon reflected in the UV region. With increasing pressure for up to 20 GPa, the maximum reflection occurred at 0 eV energy. As light travels through a medium, its intensity gradually decreases with distance. This phenomenon is known as the absorption coefficient. The figure shows that our results for the absorption coefficient and real part of optical conductivity followed a very similar pattern (Figure 19 and Figure 20). An increase in absorption causes a greater number of interactions between photons and electrons, which in turn increases the conductivity. One definition of “optical conductivity” is the amount of free charge carriers produced when photons and electrons interact.^[71]^ The presence of free charge carriers determines the structure of the optical conductivity spectra. Absorption coefficient curves are shown in Figure 20. It is true that the absorption coefficient and conductivity of all structures peak in the UV region and then drop off sharply as the energy level rises. At around 4 eV, the structures showed the greatest conductivity. Importantly, because they can't stimulate electrons, absorption and conduction don't happen at lower energies. On the contrary, they aid in the molecular oscillations. Photons in the ultraviolet‐visible spectrum can cause electrical changes. The decrease occurred when the UV region peaked because more energy transformations are induced by photons with higher energies. For absorption transitions in all materials, the X‐ray photon has a much larger size. Compared to other structures, the highest absorption coefficient value of the compound is 291234 cm−1 at 6.7 eV. There are three ways in which photons can interact with materials: reflection, transmission, and absorption. The low‐energy zone has a greater reflectivity rating since there is less absorption there. These materials have photoconductive and absorbent characteristics, which make them potential for use as UV radiation detectors, UV light coatings, and deep ultraviolet nonlinear optics.

**Figure 21:**
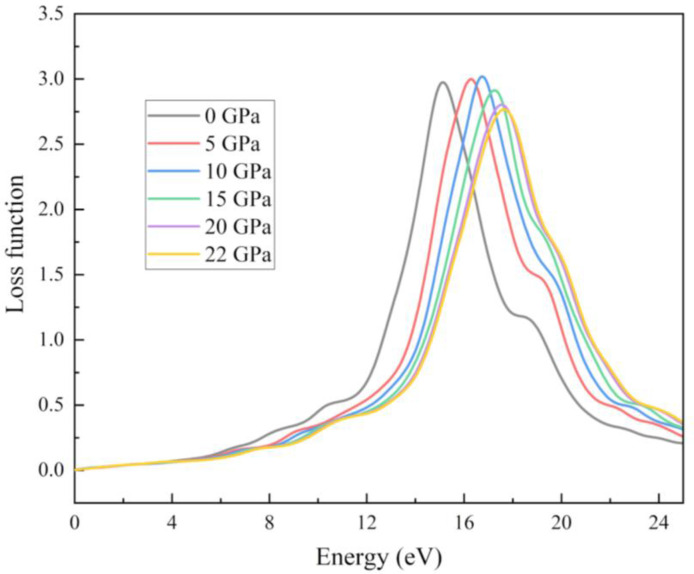
Loss function of the RbV_3_Sb_5_ structure under various applied hydrostatic pressures.

Collective excitations are also called plasmons when the plasma resonance condition is satisfied.[^72^, ^73^] This mechanism becomes quite obvious when an electron is used instead of a photon. When an electron travels through a homogeneous dielectric, its energy will drop according to the loss function. The energy loss function is constructed using the imaginary component of the dielectric function. The loss function can be expressed as$\;\left(\frac{-1}{\epsilon 2\left(\omega \right)\;}\right)$ . We obtained plasmon peak at 14.2 eV for the RbV_3_Sb_5_ structure which is given in Figure 21.

In this study we can see the impact of hydrostatic pressure on the RbV_3_Sb_5_ structure. For pristine structure maximum reflection occurred in UV region. The fact is with increasing pressure, for greater than 10 eV photon energy the maximum reflection occurred at 0 eV photon energy. For absorption coefficient, conductivity (both real and imaginary parts) and loss function graphs, similar pattern are achieved for all applied pressure. Also, the peaks of these graphs are blue shifted with increasing pressure. The obtained maximum value of real part of the refractive index at 0 GPa also increase with with increasing pressure. The peaks in the imaginary part of dielectric constant graph are red shifted as well as we obtained maximum value of imaginary part of dielectric constant for 20 Gpa.

## 3 Conclusion

DFT simulations were utilized in order to investigate the RbV_3_Sb_5_ kagome structure. Our research was carried out with the purpose of analyzing the structural, mechanical, thermal, and optoelectronic properties of the RbV_3_Sb_5_ kagome compound. According to the findings of Mulliken and Hirshfeld's population analysis, the bonding in RbV_3_Sb_5_ is comprised of both ionic and covalent connections. In accordance with the Born stability criterion, it has been established that unadulterated RbV_3_Sb_5_ demonstrates mechanical stability. With an exact value of 3.96, the machinability of our sample is much higher when subjected to a steady pressure of 20 GPa on a consistent basis. It has been demonstrated that the expected hardness of RbV_3_Sb_5_ is very low, which indicates that it has the potential to be utilized as a solid lubricant that possesses elastic qualities. For the most part, the hardness values demonstrate an upward tendency as the pressure increases; nevertheless, there may be some departures from this trend periodically. The anisotropic parameters of RbV_3_Sb_5_ display a certain degree of anisotropy, and there are also significant nonmonotonic changes that occur with pressure. Among the various pressure, RbV_3_Sb_5_ has the highest Zener anisotropic value at 20 GPa. The structure is supposed to have a Debye temperature of 284.39 K when it is subjected to 0 GPa, which indicates that it is a material that is soft. It is possible to identify the presence of structural dynamical instability in the ground state by observing dispersion curves that have frequency values that are negative. When the pressure was increased, the structure eventually reached a stable state. Although the structure is subjected to a stress of 10 GPa, it does not display any phonon branches that have negative energy. In accordance with the band structure and density of state analysis, the structure exhibits metallic behavior when subjected to a range of hydrostatic pressures. In addition, the structure demonstrates the highest conductivity, and absorption coefficients in the region of the ultra‐violet spectrum. For each and every degree of pressure that is applied, the graphs of the absorption coefficient, conductivity, and loss function all exhibit patterns that are consistent with one another. Furthermore, as the pressure increases, the peaks of these graphs display a phenomena that is referred to as blue shift.

## Conflict of Interests

All of the authors have confirmed that they have no financial or personal relationships that might be seen as influencing the work disclosed in this paper.

4

## Data Availability

Due to legal or ethical constraints, we are currently unable to provide the raw/processed data required to replicate these findings.
